# Global, regional, and national prevalence of diabetes mellitus in patients with pulmonary tuberculosis: a systematic review and meta-analysis

**DOI:** 10.1186/s13098-021-00743-3

**Published:** 2021-10-30

**Authors:** Minmin Li, Tao Chen, Zhongqiu Hua, Hong Yan, Duolao Wang, Zhaoqing Li, Yijun Kang, Ni Zhu, Chao Li

**Affiliations:** 1 Shaanxi Provincial Center for Disease Control and Prevention, No. 3 East Jian Road, PO Box 46, Xi’an, 710041 Shaanxi People’s Republic of China; 2 Department of Clinical Sciences, Liverpool School of Tropical Medicine Pembroke Place, Liverpool, UK; 3 Wuxi Early Intervention Center for Children with Special Needs, Wuxi, China; 4 Department of Epidemiology and Biostatistics, School of Public Health, Xi’an Jiaotong University Health Science Center, No. 76 West Yanta Road, PO Box 46, Xi’an, 710061 Shaanxi People’s Republic of China; 5 Nutrition and Food Safety Engineering Research Center of Shaanxi Province, Xi’an, China

**Keywords:** Diabetes mellitus, Pulmonary tuberculosis, Prevalence

## Abstract

**Background:**

Both pulmonary tuberculosis (PTB) and diabetes mellitus (DM) are major global public health problems. We estimated the global, regional, and national prevalence of diabetes mellitus in a population with PTB.

**Methods:**

We searched for observational studies of DM in people with PTB using the PubMed and Embase electronic bibliographic databases, focusing on articles published in the English language from database inception until March 31, 2021. We included original research that reported the prevalence of DM in PTB or those that had sufficient data to compute these estimates. Studies were excluded if they did not provide primary data or were case studies or reviews. Two authors independently extracted the articles and collected detailed information using a predefined questionnaire. A country-specific random-effects meta-analysis was used for countries with two or more available studies, and a fractional response regression model was employed to predict the prevalence of DM in PTB for countries with one or no study. The study was registered with the International Prospective Register of Systematic Reviews, using the registration number CRD42018101989.

**Results:**

We identified 22,658 studies, and 153, across 51 countries, were retained for data extraction. The global prevalence of DM among patients with PTB was estimated to be 13.73% (95% confidence interval [CI] 12.51–14.95). The prevalence rates were 19.32% (95% CI 13.18–25.46) in the region of the Americas, 17.31% (95% CI 12.48–22.14) in the European region, 14.62% (95% CI 12.05–17.18) in Southeast Asia, 13.59% (95% CI 7.24–19.95) in the western Pacific region, 9.61% (95% CI 4.55–14.68) in the eastern Mediterranean region, and 9.30% (95% CI 2.83–15.76) in the African region. The country with the highest estimated prevalence was the Marshall Islands (50.12%; 95% CI 4.28–95.76).

**Conclusion:**

Comorbid PTB and DM remain prevalent worldwide.

**Supplementary Information:**

The online version contains supplementary material available at 10.1186/s13098-021-00743-3.

## Background

Both tuberculosis (TB) and diabetes mellitus (DM) are major global public health problems. Despite laudable progress policies and medical care in the control of TB, the World Health Organization (WHO) reported 10 million cases of TB and 1.3 million TB-related deaths in 2017 [1]. Meanwhile, the prevalence of DM has been increasing in recent decades [2]; as of 2015, more than 415 million adults had DM, and this number is estimated to increase to 642 million by 2040 [3].

Since the early part of the twentieth century, clinicians have observed an association between DM and TB in what has been described as a co-epidemic [4–7]. Previous research has shown that the risk of developing DM is threefold higher in people with TB than in those without, suggesting that the TB epidemic is fueling the DM epidemic [8]. According to another systematic review, DM has a major effect on TB treatment outcomes in that patients with TB and DM are at greater combined risk of treatment failure, relapse, and death than those without [9]. Considering the dual burden of these two diseases, the WHO and the International Union Against Tuberculosis and Lung Disease (“the Union”) launched a collaborative framework, which emphasizes the need to establish a collaborative mechanism between National Tuberculosis Programs and diabetes organizations and to expand the bi-directional screening of TB and DM [10].

Pulmonary TB (PTB) presents in the lung, which is the major target organ in 85% of cases [11]. An increasing number of studies have demonstrated that patients with PTB are also screened for DM [12–21]. The global burden of DM among patients with TB has been estimated before [22]; however, the global prevalence of DM among patients with PTB, the most common form of TB, has not been fully determined. Therefore, we performed a systematic review and meta-analysis to estimate the global, regional, and national prevalence of DM (any type) in patients with PTB. We expect our results to provide the most up-to-date information on the national, regional, and global rates of DM among patients with PTB.

## Methods

### Protocol and registration

This review follows the Preferred Reporting Items for Systematic Reviews and Meta-Analysis (PRISMA) guidelines [23]. The full review protocol is available in the International Prospective Register of Systematic Reviews (PROSPERO), with the registration number CRD42018101989.

### Eligibility criteria

Cross-sectional studies, cohort studies, and those with monitoring data reporting the number/prevalence of DM among patients with PTB were included. We also considered case–control studies in which patients with PTB comprised the case group and that reported the prevalence or number of patients with DM in the case group. Participants of eligible studies were required to have a diagnosis of PTB.

### Information sources

Studies were identified by searching the PubMed and Embase literature databases. The search for relevant articles was limited to those that were written in English, included only human beings, and were published from database inception until March 31, 2021.

### Literature search

We developed a comprehensive systematic literature search method to standardize the screening of articles and identify all studies reporting the prevalence or number of cases of DM among patients with PTB in any country. A detailed description of the main keywords used for the search strategy is available in Additional file 1: Table S1 and Box S1, P2 and P3.

### Study selection

Two reviewers (M. M. L. and Z. Q. H.) independently selected studies for inclusion. First, the two reviewers excluded any articles that did not meet the inclusion criteria by screening titles and abstracts. Then, both reviewers read the full texts of the remaining articles. During the full-text screening, the reference lists were also reviewed to identify any additional relevant studies. Lastly, the reviewers extracted the relevant data from the included articles. If the full text of an article could not be found, we attempted to contact the corresponding author of the article to obtain the full text using the information provided in the abstract. Disagreements were resolved by team discussions about the articles retained and the data extracted.

### Data-collection process

We designed a customized questionnaire (Additional file 1: Questionnaire P5) and EpidData database for data extraction. The extracted data included the following: article title, publication time, study design, number of patients with PTB, and number or prevalence of patients with DM among the total PTB cases. Two authors (M. M. L. and Z. Q. H.) independently extracted the relevant information and input it into the EpidData version 3.1 software program (EpiData Association, Odense, Denmark). Meanwhile, we collected data on TB incidence, gross domestic product (GDP), human development index (HDI), and human capital index (HCI) from the World Bank Open Data website [24].

### Data items

The following information was extracted from each included article: (1) article title, (2) publication time, (3) study design, (4) study country, (5) study time, (6) age range, (6) number of patients with PTB, (7) number/prevalence of patients with PTB and DM. See Additional file 1: Questionnaire P4.

### Risk of bias in individual studies

The quality of each included study was assessed using a modified version of a critical appraisal tool designed for use in systematic reviews addressing questions of prevalence [25]. Two reviewers (M. M. L. and Z. Q. H.) independently assessed the studies, and any disagreement was resolved through consensus.

### Summary measures

Global, regional, and national rates of DM and PTB were the primary measures. The meta-analyses were performed by computing the prevalence rate using a random-effects model and multilevel fractional response regression modeling, and 95% confidence interval (CI) values were calculated using the Monte Carlo method.

### Planned methods of analysis

A country-specific random-effects meta-analysis method was used to estimate the pooled prevalence of DM among patients with PTB for countries with two or more empirical studies. Before performing the meta-analysis, the prevalence of DM among patients with PTB reported in each study was transformed using a double arcsine transformation. For countries with one or no empirical studies, we predicted the country’s prevalence of DM among patients with PTB using multilevel fractional response regression modeling. In detail, we generalized a linear model with a binomial family and a logit link to restrict final predictions ranging from 0 to 1. The following predictor variables were added in the fractional response regression model to predict the prevalence of comorbid PTB and DM: no predictor variables (model 1); study years and GDP in the study years (or nearest years if the GDP was unavailable in the study years) (model 2); and study years, GDP in the study years (or nearest years if GDP was unavailable in the study years), HDI for the year 2020, and HCI for the year 2020 (model 3). Data on the GDP, HDI, and HCI were acquired from the World Bank Open Data website. The standard error for each country estimate was based on the variation between studies included in the meta-analysis.

To estimate the prevalence of DM in PTB among the six WHO regions and globally, we calculated a weighted average of the prevalence of DM among patients with PTB weighted by the predicted number of patients with TB in each country for the last year with data available (2019). To estimate the 95% CI values of these point estimates, we used the Monte Carlo method (drawing 1,000,000 samples per country) to generate the corresponding 1,000,000 weighted averages of the regions and globally. The normal distribution assumption of the average was used to estimate the 95% CI values.

The *I*^2^ statistic was used to assess the heterogeneity [26]. We considered *I*^2^ values of 25% to 49% as indicative of low heterogeneity, 50% to 74% as indicative of moderate heterogeneity, and at least 75% as indicative of high heterogeneity, respectively [27]. All analyses were performed using Stata version 15.0 (StataCorp LLC, College Station, TX, USA) [28].

### Risk of bias across studies

We used Egger’s test to detect publication bias [14]. A *P*-value of less than 0.10 on Egger’s test was considered indicative of statistically significant publication bias. It was decided a priori that, if publication bias were present, it would not be adjusted for, given that it was assumed that the prevalence estimates of interest would likely be published even if they were substantially different from previously reported estimates.

## Results

### Study selection

**Fig. 1:**
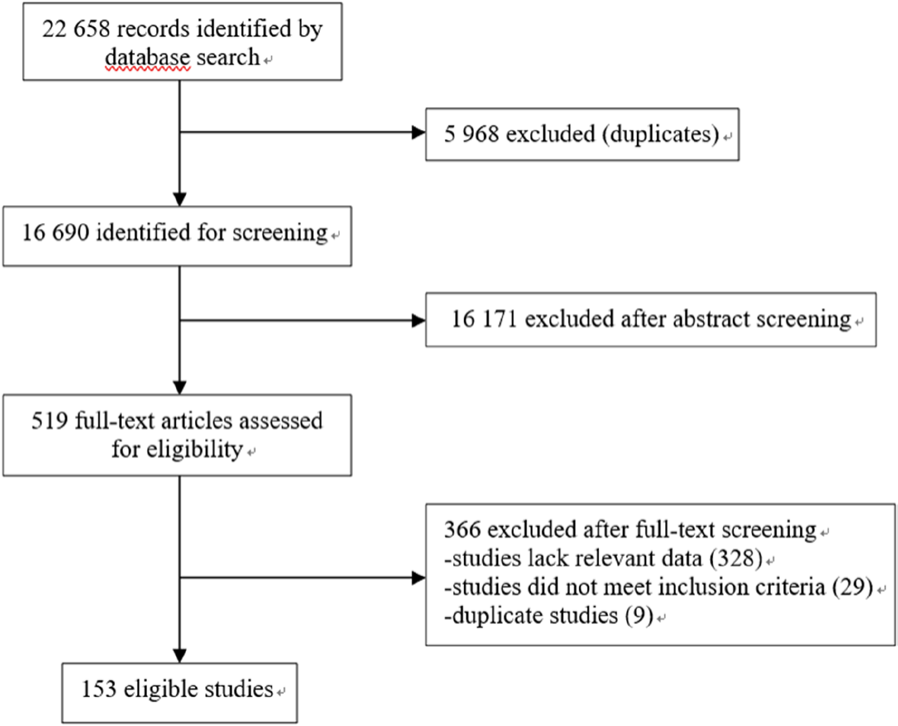
Flow chart of literature search for studies on the prevalence of DM in patients with PTB

The search of the PubMed and Embase databases provided a total of 22,658 citations. After adjusting for duplicates, 16,690 remained. Of these, 16,171 studies were further discarded after their abstracts were read because it appeared that these papers clearly did not meet the criteria, and the full texts of the remaining 519 citations were examined in greater detail; at this point, it appeared that another 366 studies did not meet the inclusion criteria as described. Finally, a total of 153 studies were identified for inclusion in the present review. No unpublished relevant studies were obtained (Fig. 1).

### Study characteristics

All 153 articles finally selected for this review were observational studies published in the English language before March 31, 2021. The included studies contained 983,552 patients with PTB, including 138,474 with DM, across 51 countries. Data were available for 17 studies involving 13 countries in the African region; 15 studies involving six countries in the eastern Mediterranean region; 12 studies involving nine countries in the European region; 33 studies involving seven countries in the region of the Americas; 40 studies involving eight countries in the Southeast Asian region; and 39 studies involving eight countries in the western Pacific region. Twenty countries had two or more studies, and the three countries with the most studies were China (*n* = 22), India (*n* = 20), and Mexico (*n* = 9).

### Results of individual studies

The prevalence of DM among patients with PTB in the included articles is shown in Additional file 1: Table S2.

### Risk of bias within studies

The critical appraisal of included studies and a complete reference list of all included studies are shown in Additional file 1: Table S3.

### Syntheses of results

The prevalence of DM among patients with PTB was estimated for 184 countries (via a meta-analysis for 20 countries [with two or more available empirical studies] and via statistical modeling [prediction] for 164 countries). The prevalence of DM among patients with PTB could not be estimated for 11 countries because of missing data for one or more predictor variables. The final model included the following predictor variables: GDP (β = 0.01 [referring to an increase of 100,000 international dollars]; 95% CI − 0.01 to 0.01), HDI (β = 0.24 [referring to a 10% increase]; 95% CI − 0.96 to 1.43), TB incidence (β =  − 0.01 [referring to a 1% increase]; 95% CI − 0.01 to 0.01), and DM prevalence (β = 0.09 [referring to a 1% increase]; 95% CI − 0.14 to 0.31).

**Fig. 2:**
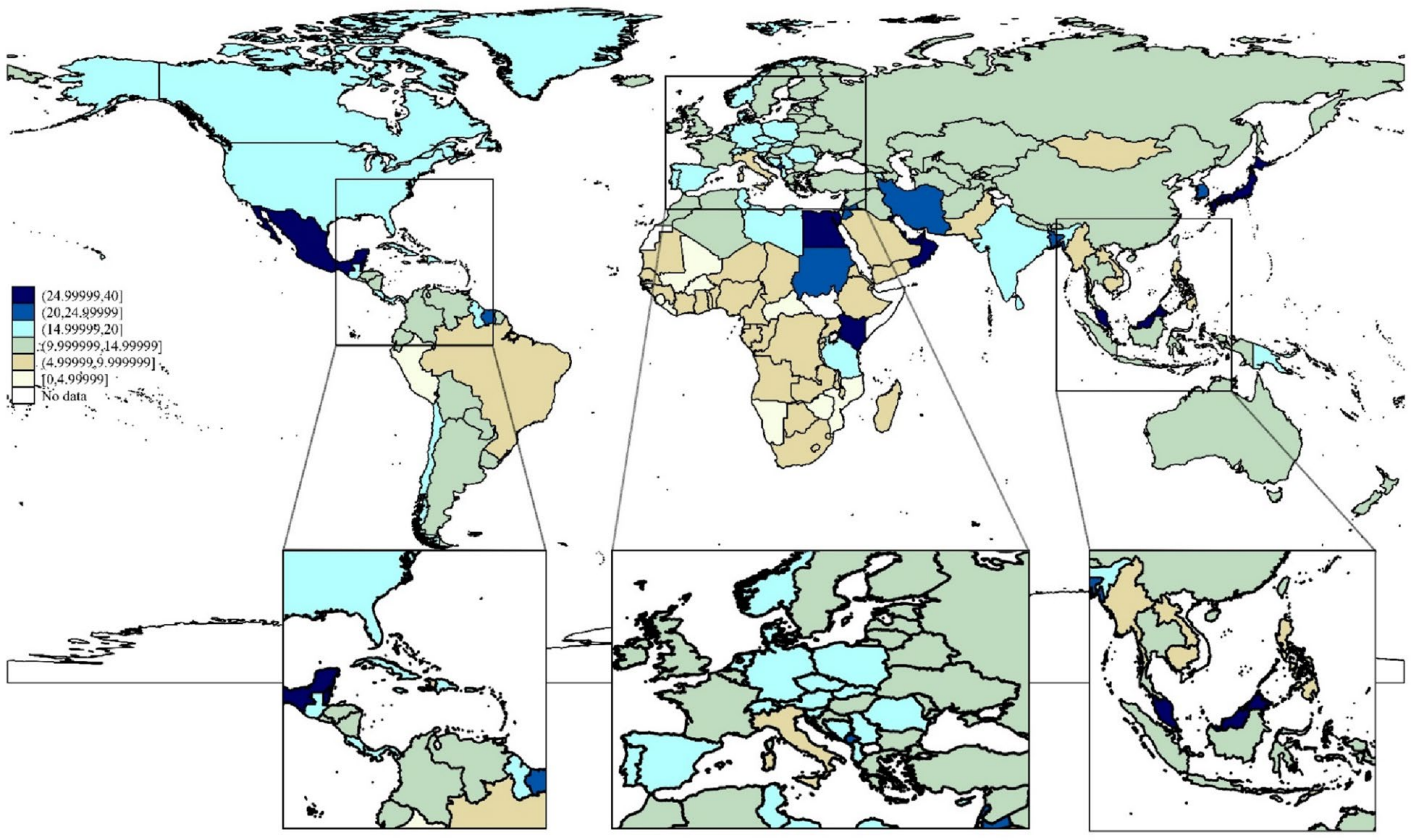
Global prevalence of PTB combined DM. A multilevel fractional response regression modeling (for countries with one or no empirical studies) and country-specific random- effects meta-analysis methods (for countries with two or more empirical studies) were used to predict the country’s prevalence of DM in PTB patients

The five countries with the highest estimated prevalence of DM among patients with PTB were the Marshall Islands (50.12%; 95% CI 4.28–95.76), Mauritius (41.59%; 95% CI 4.02–92.38), Nauru (40.89%; 95% CI 1.80–96.31), the United Arab Emirates (36.42%; 95% CI 5.07–79.31), and Bahrain (32.81%; 95% CI 5.86–79.32). The five countries with the lowest prevalence of DM among patients with PTB were Lesotho (3.90%; 95% CI 0.04–782.06), Mozambique (4.01%; 95% CI 0.07–72.62), Liberia (4.36%; 95% CI 0.18–52.57), Sierra Leone (4.43%; 95% CI 0.21–50.39), and the Central African Republic (4.53%; 95% CI 0.09–70.93). The prevalence of DM among patients with PTB by country is illustrated in Fig. 2 and Additional file 1: Table S4. The results of the tests of heterogeneity and publication bias for the meta-analysis of the prevalence of DM among patients with PTB by country are shown in Additional file 1: Table S5.

The global prevalence of DM among patients with PTB was estimated to be 13.73% (95% CI 12.51–14.95). The highest prevalence of DM among patients with PTB was in the region of the Americas (19.32%; 95% CI 13.18–25.46), and the lowest prevalence of DM among patients with PTB was in the African region (9.30%; 95% CI 2.83–15.76). Additionally, the prevalence of DM among patients with PTB was 17.31% (95% CI 12.48–22.14) in the European region, 14.62% (95% CI 12.05–17.18) in the Southeast Asian region, 13.59% (95% CI 7.24–19.95) in the western Pacific region, and 9.61% (95% CI 4.55–14.68) in the eastern Mediterranean region (Table 1).

**Table 1:** The global prevalence (%) of DM in patients with PTB, by country and WHO region

WHO Region	Prevalence (%)	95% confidence interval	
		Lower	Upper
African Region	9.30	2.83	15.76
Eastern Mediterranean Region	9.61	4.55	14.68
European Region	17.31	12.48	22.14
Region of the Americas	19.32	13.18	25.46
South-East Asia region	14.62	12.05	17.18
Western Pacific Region	13.59	7.24	19.95
Globally	13.73	12.51	14.95

95% CI were calculated by using the Monte Carlo method

*WHO* world health organization

## Discussion

### Summary of evidence

In the present study, we estimated the global, regional, and national prevalence rates of DM among patients with PTB for the first time. Although the prevalence of PTB appears to be decreasing overall, comorbid PTB and DM remain prevalent in several countries. First, we found that the global predicted prevalence of DM among patients with PTB was 13.73% (95% CI 12.51–14.95). Additionally, the prevalence among individual regions ranged from 9.30% (95% CI 2.83–15.76) in the African region to 19.32% (95% CI 13.18–25.46) in the region of the Americas, and the prevalence among countries ranged from 3.90% (95% CI 0.04–82.06) in Lesotho to 50.12% (95% CI 4.28–95.76) in the Marshall Islands.

### Differences in prevalence rates

Compared with the global prevalence, the prevalence of DM in patients with PTB was higher in the region of the Americas, the European region, and the Southeast Asia region, while the African region had the lowest prevalence of comorbid PTB and DM. This may be linked to the fact that countries in the region of the Americas have experienced the fastest increase in DM prevalence along with a high burden of TB and human immunodeficiency virus [29, 30]. The European region also showed a high prevalence of DM among patients with PTB, which can be attributed to effective infection disease prevention and better-resourced health systems [31]. India has the highest number of TB cases (27%) in the world and a very high burden of DM, even within the Southeast Asian region [32]. The African region has the lowest prevalence of DM, which could be explained by the lack of some DM risk factors, including prevalence of excess weight and obesity, aging of the population, and hypertension [18]. In addition, most PTB and DM cases in African regions are not registered at the local department, and the majority of facilities are still not screening for DM among patients with PTB, partly due to cost and perceived complexities. Furthermore, there is a lack of treatment infrastructure for those who do screen positive, which can lead to underdiagnosis of DM, which may also explain the underprevalence of DM among patients with PTB in the African region [33–36].

The five countries with the highest estimated prevalence of comorbid PTB and DM were the Marshall Islands, Mauritius, Nauru, the United Arab Emirates, and Bahrain. The five countries with the lowest prevalence of comorbid PTB and DM were Lesotho, Mozambique, Liberia, Sierra Leone, and the Central African Republic. Sociodemographic factors (sex, age, and income), behavioral attributes (tobacco smoking and alcohol drinking), and clinical and other factors are also associated with comorbid TB and DM [1]. The differences in prevalence rates of these conditions between countries can also be explained by variations in the risk factors of comorbid PTB and DM.

Sensitivity analyses revealed high heterogeneity in Brazil. Several factors may have contributed to this heterogeneity, including the sampling methodology, study subjects, study year(s), geographical location, variability within the specific subpopulation studied, sex and age-group representation in the population sample, and publication bias. However, it was not possible to quantify the contribution of these sources of variation to the heterogeneity in the association through a meta-regression analysis given the relatively small number of studies available.

Numerous studies have presented convincing biological evidence in support of the causal relationship between DM and impaired host immunity to TB. Indeed, several recent animal models of *Mycobacterium tuberculosis* have demonstrated an unexpected development of DM, particularly in those treated with anti-glycemic therapy as host-directed therapy for TB [37]. Thus, PTB may identify individuals at higher risk of progression to DM. Another possible mechanism by which PTB may increase the risk of DM is through changes in body composition during and following the illness. Patients with PTB frequently lose a substantial amount of weight before and in the early stages of treatment. Limited evidence from cohort studies suggests that the regaining of weight during treatment could increase the proportion of body fat in recovered patients with TB, which may increase their future risk of DM [38, 39].

### Public health recommendations

Given the high prevalence of comorbid PTB and DM and its adverse outcomes, we strongly believe that greater investments are needed to reduce the prevalence of comorbid PTB and DM. There are several recommendations to facilitate such improvements: for the Department of Public Health, conduct early bi-directional DM screening for patients with PTB, especially in the African region, as well as improving collection and monitoring of data for PTB and DM; for clinical medicine organizations, focus on developing and implementing clinical guidelines and tools to improve the management and care of individuals with PTB at risk of DM; and, for the medical research institutions, perform further studies to understand whether DM causes PTB or vice versa so that this combination can be better predicted and prevented.

## Limitations

There are several potential limitations to this study. First, we did not include studies published in all languages, which may have resulted in some relevant studies being excluded. However, as high-quality studies tend to be published in English, we do not consider this to have had a significant impact on our findings. Second, we included studies that did not consistently define the diagnosis of PTB and DM, with different criteria being used in different countries. However, the diagnosis of both PTB and DM in most of the countries references the WHO standards. Third, the predicted prevalence estimates for the 168 countries with either one or no available study might diverge from the actual prevalence because the data from which the values were predicted carry some measurement error. In addition, other relevant explanatory variables, which may not be possible to account for, may affect the prevalence of DM with PTB. However, taking into consideration this study and the fact that we were limited to the information reported in the included studies, we consider the present model to have yielded the best estimates. Finally, it should also be noted that this study was limited to WHO member states.

## Conclusions

In conclusion, comorbid PTB and DM is a crucial global health issue that must be addressed to reduce adverse treatment outcomes and mortality globally and improve patients’ quality of life. Our findings suggest that early standardized bi-directional screening of DM in patients with TB and screening of TB in patients with DM should be implemented as soon as possible. DM control programs should also consider targeting patients with PTB for interventions, such as active case finding and the treatment of hyperglycemia. Efforts to diagnose, detect, and treat PTB may have a beneficial impact on DM control. To better understand the global epidemiology of DM among patients with PTB, the quality and volume of data need to be strengthened, including standardizing definitions, measurement, monitoring, and reporting. Further research on the causes of comorbid PTB and DM and new interventions to prevent and manage the consequences of comorbid PTB and DM (particularly in low- and middle-income regions) are also needed.

## Supplementary Information

**Additional file 1.** **Table S1**. Electronic bibliographic databases and keywords used in the comprehensive systematic literature search. **Box S1**. Search strategy to identify studies on the association of pulmonary tuberculosis and diabetes mellitus. Questionnaire. The questionnaire of national, regional, and global prevalence of diabetes mellitus in patients with pulmonary tuberculosis. **Table S2**. Sample size of the included studies and the reported prevalence of diabetes mellitus in patients with pulmonary tuberculosis, by country and WHO region. **Table S3**. Result of the quality appraisal of the included studies. **Table S4**. The estimated prevalence of diabetes mellitus in patients with pulmonary tuberculosis, by country and WHO region. **Table S5**. Results of the test of heterogeneity and publication bias for the meta-analysis of the prevalence of diabetes mellitus in patients with pulmonary tuberculosis, by country and WHO region.

## Data Availability

The dataset supporting the conclusions of this article is included within Additional file 1.

## Abbreviations

CI: Confidence interval
DM: Diabetes mellitus
GDP: Gross domestic product
HCI: Human capital index
HDI: Human development index
PTB: Pulmonary tuberculosis
TB: Tuberculosis
WHO: World Health Organization
