# Supplementary material for: Global, regional, and national prevalence of diabetes mellitus in patients with pulmonary tuberculosis: a systematic review and meta-analysis
Source: Diabetol Metab Syndr. 2021 Oct 30;13:127. doi: 10.1186/s13098-021-00743-3 (PMC8557479; doi:10.1186/s13098-021-00743-3)
Supplement: Supplementary file 1 — Additional file 1. Table S1. Electronic bibliographic databases and keywords used in the comprehensive systematic literature search. Box S1. Search strategy to identify studies on the association of pulmonary tuberculosis and diabetes mellitus. Questionnaire. The questionnaire of national, regional, and global prevalence of diabetes mellitus in patients with pulmonary tuberculosis. Table S2. Sample size of the included studies and the reported prevalence of diabetes mellitus in patients with pulmonary tuberculosis, by country and WHO region. Table S3. Result of the quality appraisal of the included studies. Table S4. The estimated prevalence of diabetes mellitus in patients with pulmonary tuberculosis, by country and WHO region. Table S5. Results of the test of heterogeneity and publication bias for the meta-analysis of the prevalence of diabetes mellitus in patients with pulmonary tuberculosis, by country and WHO region. [file 13098_2021_743_MOESM1_ESM.doc]

**ADDITIONAL FILE 1**

**Global, regional, and national prevalence of diabetes mellitus in patients with pulmonary tuberculosis: a systematic review and meta-analysis**

**TABLE OF CONTENS**

Table S1 2

Box S1 3

Questionnaire 4

Table S2 5

Table S3 9

Table S4 17

Table S5 23

References 24

**Table S1. Electronic bibliographic databases and keywords used in the comprehensive systematic literature search**

| Electronic bibliographic databases | Pubmed and Embase |
| --- | --- |
| Keywords | 1) tuberculosis OR TB OR pulmonary tuberculosis; AND  2) diabetes mellitus OR DM OR glucose intolerance OR glucose tolerance OR insulin resistance OR hyperglycaemia; AND  3) chronic disease OR non-communicable disease; AND  4) observational study OR cohort studies OR cross-sectional study OR epidemiologic studies OR retrospective studies OR follow-up studies OR longitudinal studies |

**Box S1.** Search strategy to identify studies on the association of pulmonary tuberculosis and diabetes mellitus

**Pubmed**

MESH terms

1. Tuberculosis (TB) OR pulmonary tuberculosis (PTB)
2. diabetes mellitus (DM)
3. cross-sectional study OR cohort studies OR epidemiologic studies OR observational study OR retrospective studies OR follow-up studies OR longitudinal studies

TEXT terms

1. tuberculosis OR pulmonary tuberculosis
2. diabetes mellitus OR DM OR glucose intolerance OR glucose tolerance OR insulin resistance OR hyperglycaemia
3. chronic disease OR non-communicable disease

Search string (all inclusive)

1. 1 AND 2
2. 1 AND 3 AND 5
3. 1 AND 3 AND 6
4. 4 AND 5

**EMBASE**

TEXT terms

1. tuberculosis OR pulmonary tuberculosis
2. diabetes mellitus
3. cross-sectional study OR cohort studies OR epidemiologic studies OR observational study OR retrospective studies OR follow-up studies OR longitudinal studies

Search string (all inclusive)

1. 1 AND 2
2. 1 AND 3

**Questionnaire**

The questionnaire of national, regional, and global prevalence of diabetes mellitus in patients with pulmonary tuberculosis

1. Code:
2. Title:
3. Published time:
4. Study design:
5. Study region:
6. Study time:
7. Number of PTB:
8. Age range:________
9. Number of PTB with DM or prevalence:
10. Was the sample representative sample of the target population (PTB)**:**  ①YES ②NO
11. Were the study population recruited in an appropriate way: ①YES ②NO
12. Was the sample size adequate(n>300): ①YES ②NO
13. Were the participants and setting described in detail: ①YES ②NO
14. Is sufficient coverage of the identified sample: ①YES ②NO
15. Were objective criteria used for ascertaining DM: ①YES ②NO
16. Was there appropriate statistical analysis: ①YES ②NO

**Table S2.** Sample size of the included studies (n=153) and the reported prevalence of diabetes mellitus in patients with pulmonary tuberculosis, by country and WHO region

| **Country** | **Reference** | **Sample size** | **Prevalence（%）** |
| --- | --- | --- | --- |
| **AFRICAN REGION (17)** | | | |
| Angola | Segafredo et al 128 | 7025 | 6.1 |
| Cameroon | Tchankam et al 9 | 347 | 4.3 |
| Ethiopia | Workneh et al 33 | 770 | 9.1 |
| Eritrea | Araia et al1 29 | 751 | 11.1 |
| Ghana | Yorke et al 39 | 146 | 11.6 |
| Guinea | Haraldsdottir et al 40 | 110 | 2.7 |
| Kenya | Owiti et al 74 | 389 | 4.4 |
| Kenya | Mburu et al 75 | 347 | 37.2 |
| Mozambique | Pizzol et al 94 | 301 | 1.0 |
| Nigeria | Ogbera et al 97 | 3376 | 4.8 |
| Nigeria | Ekeke et al 98 | 1996 | 9.5 |
| Nigeria | Ogbera et al 99 | 4000 | 12.0 |
| South Africa | Grint et al 66 | 259 | 6.9 |
| Tanzania | Faurholt-Jepsen et al 112 | 1205 | 16.3 |
| Tanzania | Faurholt-Jepsen et al 113 | 803 | 16.7 |
| Uganda | Kibirige et al 117 | 197 | 9.6 |
| Zambia | Fwoloshi et al 127 | 127 | 4.7 |
| **EASTERN MEDITERNEAN REGION (15)** | | | |
| Egypt | Hasanain et al 31 | 231 | 27.7 |
| Egypt | Gadallah, M., et al130 |  |  |
| Iran | Alavi-Naini et al 67 | 715 | 15.1 |
| Iran | Golsha et al 68 | 243 | 23.0 |
| Iran | Alavi et al 69 | 148 | 24.3 |
| Kuwait | Abal et al 82 | 526 | 29.8 |
| Pakistan | Aftab et al 100 | 2808 | 39.6 |
| Pakistan | Mukhtar et al 101 | 614 | 18.4 |
| Pakistan | Jawad et al 102 | 106 | 19.8 |
| Pakistan | Aftab et al 103 | 268 | 4.9 |
| Pakistan | Hasan et al131 | 172 | 14.0 |
| Pakistan | Hameed et al132 | 170 | 18.8 |
| Qatar | Al-Shaer et al 106 | 148 | 22.3 |
| Saudi Arabia | Alkabab et al 107 | 133 | 28.6 |
| Saudi Arabia | Chaudhry et al 108 | 1388 | 8.2 |
| **EUROPEAN REGION (12)** | | | |
| Armenia | Sahakyan et al 133 | 621 | 6.0 |
| France | Delory et al 37 | 177 | 13.0 |
| Georgia | Salindri et al 38 | 268 | 13.4 |
| Italy | Caraffa et al 70 | 857 | 6.3 |
| Italy | Sane et al 134 | 910 | 25.1 |
| Kazakhstan | Hermosilla et al 73 | 562 | 7.1 |
| Romania | Grint et al 66 | 469 | 6.6 |
| Spain | Moreno-Martinez et al 110 | 4250 | 5.9 |
| Turkey | Bacakoglu et al 115 | 927 | 9.9 |
| Turkey | Guler et al 116 | 306 | 14.4 |
| United Kingdom | Walker et al 118 | 3461 | 11.1 |
| United Kingdom | Kreisel et al135 | 55 | 45.4 |
| **REGION OF THE AMERICAS (33)** | | | |
| Argentina | Fernández et al 1 | 35 | 40.0 |
| Brazil | Picon et al 2 | 610 | 8.9 |
| Brazil | Gomes et al 3 | 300675 | 7.1 |
| Brazil | Moreira et al 4 | 211 | 2.8 |
| Brazil | Augusto et al 5 | 41642 | 3.2 |
| Brazil | Leal et al 6 | 323 | 14.6 |
| Brazil | Reis-Santos et al 7 | 25047 | 6.6 |
| Brazil | Pereira et al 8 | 323 | 13.6 |
| Guyana | Alladin et al 41 | 90 | 15.6 |
| Kiribati | Cavanaugh et al 76 | 218 | 37.2 |
| Mexico | Blanco-Guillot et al 85 | 1370 | 33.0 |
| Mexico | Perez-Navarro et al 86 | 507 | 36.1 |
| Mexico | Chittoor et al 87 | 75 | 49.3 |
| Mexico | Jiménez-Corona et al 88 | 1262 | 29.6 |
| Mexico | Castellanos-Joya et al 89 | 361 | 19.4 |
| Mexico | Munoz-Torrico et al 90 | 90 | 54.4 |
| Mexico | Abdelbary et al 91 | 7754 | 26.4 |
| Mexico | Ponce-De-Leon et al 92 | 581 | 29.6 |
| Mexico | Delgado et al 93 | 181378 | 19.3 |
| Mexico | Rashak et al | 4954 | 20.4 |
| Peru | Byrne et al 104 | 176 | 11.9 |
| Peru | Magee et al 105 | 1633 | 11.2 |
| Peru | Grint et al 66 | 562 | 3.0 |
| Unite States | Restrepo et al 119 | 233 | 36.9 |
| Unite States | Dooley et al 120 | 228 | 16.7 |
| Unite States | Magee et al 121 | 318 | 11.6 |
| Unite States | Qian et al 122 | 7901 | 15.5 |
| Unite States | Alkabab et al 123 | 743 | 16.0 |
| Unite States | Suwanpimolkul et al 124 | 692 | 17.1 |
| Unite States | Magee et al 125 | 1067 | 12.2 |
| Unite States | Magee et al 126 | 1852 | 4.6 |
| Unite States | Nguyen et al 136 | 7727 | 17.4 |
| Unite States | Pang et al 137 | 12846 | 3.3 |
| **SOUTH-EAST ASIA REGION (40)** | | | |
| Bangladesh | Sarker et al 32 | 1570 | 13.2 |
| Bangladesh | Paul et al 138 | 1046 | 24.1 |
| Brunei Darussalam | Omar et al 139 | 1098 | 37.2 |
| India | Pande et al 42 | 406 | 35.0 |
| India | Siddiqui et al 43 | 191 | 19.4 |
| India | Balakrishnan et al 44 | 344 | 48.8 |
| India | Viswanathan et al 45 | 547 | 27.4 |
| India | Dave et al 46 | 482 | 7.1 |
| India | KV et al 47 | 2239 | 25.2 |
| India | Nair et al 48 | 567 | 38.1 |
| India | Prakash et al 49 | 205 | 14.6 |
| India | Mehta et al 50 | 194 | 11.3 |
| India | Manjareeka et al 51 | 101 | 13.9 |
| India | Mave et al 52 | 890 | 18.2 |
| India | Veesa et al 53 | 880 | 20.0 |
| India | Venkatarathnamma et al 54 | 245 | 15.9 |
| India | Gupte et al 55 | 392 | 19.1 |
| India | Viswanathan 56 | 332 | 28.9 |
| India | Naik et al 57 | 250 | 17.2 |
| India | Mahishale et al 58 | 1935 | 40.0 |
| India | Gupte et al 59 | 192 | 31.8 |
| India | Khanna et al 60 | 286 | 19.2 |
| India | Kumar et al 61 | 1241 | 30.3 |
| India | Sharma et al 140 | 157 | 20.4 |
| India | Krishnappa et al 141 | 200 | 10.5 |
| India | Deshmukh et al 142 | 2359 | 20.5 |
| India | Majumdar et al 143 | 400 | 24.5 |
| India | Christopher et al 144 | 172 | 16.3 |
| India | Nagar et al 145 | 401 | 14.5 |
| India | Sembiah et al 146 | 462 | 15.4 |
| India | Kodiatte et al 147 | 159 | 40.2 |
| Indonesia | Saktiawati et al 62 | 356 | 6.5 |
| Indonesia | Alisjahbana et al 63 | 634 | 14.8 |
| Indonesia | Stalenhoef et al 64 | 71 | 32.4 |
| Indonesia | Fachri et al 65 | 225 | 36.0 |
| Indonesia | Grint et al 66 | 649 | 5.2 |
| Myanmar | Khan 95 | 393 | 7.9 |
| Nepal | Sreeramareddy et al 96 | 244 | 6.6 |
| Sri Lanka | Rajapakshe et al 111 | 85 | 11.8 |
| Thailand | Duangrithi et al 114 | 310 | 16.5 |
| **WESTERN PACIFIC REGION (39)** | | | |
| China | Chang et al 10 | 159566 | 23.9 |
| China | Zhao et al 11 | 283 | 42.4 |
| China | Mi et al 12 | 1578 | 11.9 |
| China | Wang et al 13 | 6382 | 6.3 |
| China | Wu et al 14 | 201 | 19.9 |
| China | Ma et al 15 | 1313 | 12.0 |
| China | Huang et al 16 | 1508 | 2.2 |
| China | Wang et al 17 | 2280 | 6.9 |
| China | Wang et al 18 | 305 | 29.8 |
| China | Wang et al 19 | 461 | 21.5 |
| China | Wang et al 20 | 157 | 32.1 |
| China | Zhao et al 21 | 1252 | 7.7 |
| China | Zhang et al 22 | 1896 | 8.5 |
| China | Mi et al 23 | 621 | 30.1 |
| China | Leung et al 24 | 462 | 19.5 |
| China | Cai et al 25 | 3505 | 2.7 |
| China | Wang et al 26 | 154 | 31.2 |
| China | Ko et al 27 | 9067 | 26.7 |
| China | Tsao et al 28 | 884 | 19.1 |
| China | Tsao et al 28 | 942 | 14.5 |
| China | Chang et al 29 | 438 | 29.5 |
| China | Leung et al 30 | 18414 | 16.6 |
| China | Wan et al | 6759 | 6.3 |
| China | Argita et al | 12477 | 17.2 |
| Fiji | Prasad et al 34 | 432 | 14.1 |
| Fiji | Gounder et al 35 | 107 | 13.1 |
| Fiji | Alo et al 36 | 326 | 15.0 |
| Japan | Nakamura et al 71 | 260 | 26.5 |
| Japan | Uchimura et al 72 | 75998 | 13.4 |
| Korea | Lee et al 77 | 499 | 21.0 |
| Korea | Lee et al 78 | 1044 | 24.2 |
| Korea | Yoon et al 79 | 661 | 23.8 |
| Korea | Park et al 80 | 492 | 25.2 |
| Korea | Choi et al 81 | 669 | 22.3 |
| Korea | Lee et al 148 | 499 | 21.0 |
| Malaysia | Sulaiman et al 83 | 1092 | 28.2 |
| Marshall Island | Nassa et al 84 | 57 | 47.4 |
| Singapore | Loh et al 109 | 75 | 30.7 |
| Vietnam | Hoa et al 149 | 662 | 18.1 |

**Table S3.** Result of the quality appraisal of the included studies (n=153)

| Reference | 1.Representative sample of the target population (PTB) | 2.Appropriate  recruitment of participants | 3.Sample size calculate  (N>300) | 4.Detalited description of participants and setting | 5.Sufficient coverage of the identified sample | 6.Use of objective  criteria for ascertaining  DM status | 7.Appropriate statistical analysis |
| --- | --- | --- | --- | --- | --- | --- | --- |
| Abal et al^82^ | **YES** | **YES** | **YES** | **YES** | **YES** | **YES** | **YES** |
| Abdelbary et al^91^ | **YES** | **YES** | **YES** | **YES** | **YES** | **YES** | **YES** |
| Lee et al^148^ | **YES** | **YES** | **YES** | **YES** | **YES** | **YES** | **YES** |
| Aftab et al^100^ | **NO** | **YES** | **NO** | **YES** | **NO** | **YES** | **YES** |
| Aftab et al^103^ | **NO** | **YES** | **NO** | **YES** | **YES** | **YES** | **YES** |
| Alavi et al^69^ | **YES** | **YES** | **NO** | **YES** | **YES** | **YES** | **YES** |
| Alavi-Naini et al^67^ | **YES** | **YES** | **YES** | **YES** | **YES** | **YES** | **YES** |
| Alisjahbana et al^63^ | **YES** | **YES** | **YES** | **YES** | **YES** | **YES** | **YES** |
| Alkabab et al^107^ | **YES** | **YES** | **NO** | **YES** | **YES** | **YES** | **YES** |
| Alkabab et al^123^ | **YES** | **YES** | **YES** | **YES** | **YES** | **YES** | **YES** |
| Alladin et al^41^ | **YES** | **YES** | **NO** | **YES** | **YES** | **YES** | **YES** |
| Al-Shaer et al^106^ | **YES** | **YES** | **NO** | **YES** | **NO** | **NO** | **YES** |
| Alo et al^36^ | **YES** | **YES** | **YES** | **YES** | **YES** | **YES** | **YES** |
| Augusto et al^5^ | **YES** | **YES** | **YES** | **YES** | **NO** | **NO** | **YES** |
| Bacakoglu et al^115^ | **YES** | **YES** | **YES** | **YES** | **YES** | **YES** | **YES** |
| Balakrishnan et al^44^ | **YES** | **YES** | **YES** | **YES** | **YES** | **YES** | **YES** |
| Blanco-Guillot et al^85^ | **YES** | **YES** | **YES** | **YES** | **YES** | **YES** | **YES** |
| Byrne et al^104^ | **YES** | **YES** | **NO** | **YES** | **NO** | **NO** | **YES** |
| Cai et al^25^ | **YES** | **YES** | **YES** | **YES** | **YES** | **YES** | **YES** |
| Caraffa et al^70^ | **YES** | **YES** | **YES** | **YES** | **YES** | **YES** | **YES** |
| Castellanos-Joya et al^89^ | **YES** | **YES** | **YES** | **YES** | **YES** | **YES** | **YES** |
| Cavanaugh et al^76^ | **YES** | **YES** | **NO** | **YES** | **YES** | **YES** | **YES** |
| Chang et al^10^ | **YES** | **YES** | **YES** | **YES** | **NO** | **NO** | **YES** |
| Chang et al^29^ | **YES** | **YES** | **YES** | **YES** | **YES** | **YES** | **YES** |
| Kreisel et al^135^ | **YES** | **YES** | **YES** | **YES** | **YES** | **YES** | **YES** |
| Chaudhry et al^108^ | **YES** | **YES** | **YES** | **NO** | **NO** | **NO** | **YES** |
| Chittoor et al^87^ | **YES** | **YES** | **NO** | **YES** | **NO** | **NO** | **YES** |
| Choi et al^81^ | **YES** | **YES** | **YES** | **YES** | **NO** | **NO** | **YES** |
| Krishnappa et al^141^ | **YES** | **YES** | **YES** | **YES** | **YES** | **YES** | **YES** |
| Dave et al^46^ | **YES** | **YES** | **YES** | **YES** | **YES** | **YES** | **YES** |
| Sharma et al^140^ | **YES** | **YES** | **NO** | **YES** | **YES** | **YES** | **YES** |
| Delgado-Sánchezet al^93^ | **YES** | **YES** | **YES** | **YES** | **YES** | **YES** | **YES** |
| Delory et al^37^ | **YES** | **YES** | **NO** | **YES** | **YES** | **NO** | **YES** |
| Deshmukh et al^142^ | **YES** | **YES** | **YES** | **YES** | **YES** | **YES** | **YES** |
| Christopher et al^144^ | **YES** | **YES** | **YES** | **YES** | **YES** | **YES** | **YES** |
| Dooley et al^120^ | **YES** | **YES** | **NO** | **YES** | **YES** | **NO** | **YES** |
| Duangrithi et al^114^ | **YES** | **YES** | **YES** | **YES** | **YES** | **YES** | **YES** |
| Nguyen et al^136^ | **YES** | **YES** | **YES** | **YES** | **YES** | **YES** | **YES** |
| Ekeke et al^98^ | **YES** | **YES** | **YES** | **YES** | **YES** | **YES** | **YES** |
| Fachri et al^65^ | **YES** | **YES** | **NO** | **YES** | **YES** | **YES** | **YES** |
| Faurholt-Jepsen et al^112^ | **YES** | **YES** | **YES** | **YES** | **YES** | **YES** | **YES** |
| Faurholt-Jepsen et al^113^ | **YES** | **YES** | **YES** | **YES** | **YES** | **YES** | **YES** |
| Fernández et al^1^ | **NO** | **YES** | **NO** | **YES** | **YES** | **YES** | **YES** |
| Fwoloshi et al^127^ | **NO** | **YES** | **NO** | **YES** | **YES** | **YES** | **YES** |
| Segafredo et al^128^ | **YES** | **YES** | **YES** | **YES** | **YES** | **YES** | **YES** |
| Golsha et al^68^ | **YES** | **YES** | **NO** | **YES** | **NO** | **YES** | **YES** |
| Gomes et al^3^ | **YES** | **YES** | **YES** | **YES** | **NO** | **NO** | **YES** |
| Gomes et al^34^ | **YES** | **YES** | **YES** | **YES** | **YES** | **YES** | **YES** |
| Gounder et al^35^ | **YES** | **YES** | **NO** | **YES** | **YES** | **YES** | **YES** |
| Grint et al^66^ | **YES** | **YES** | **YES** | **YES** | **YES** | **YES** | **YES** |
| Guler et al^116^ | **YES** | **YES** | **YES** | **YES** | **NO** | **NO** | **YES** |
| Gupte et al^59^ | **YES** | **YES** | **NO** | **YES** | **YES** | **NO** | **YES** |
| Gupte et al^55^ | **YES** | **YES** | **YES** | **YES** | **YES** | **YES** | **YES** |
| Haraldsdottir et al^40^ | **YES** | **YES** | **NO** | **YES** | **YES** | **YES** | **YES** |
| Hasanain et al^31^ | **YES** | **YES** | **NO** | **YES** | **NO** | **NO** | **YES** |
| Hermosilla et al^73^ | **YES** | **YES** | **YES** | **YES** | **NO** | **NO** | **YES** |
| Hoa et al^149^ | **YES** | **YES** | **YES** | **YES** | **YES** | **YES** | **YES** |
| Huang et al^16^ | **YES** | **YES** | **YES** | **YES** | **NO** | **NO** | **YES** |
| Jawad et al^102^ | **YES** | **YES** | **NO** | **NO** | **NO** | **YES** | **YES** |
| Jiménez-Corona et al^88^ | **YES** | **YES** | **YES** | **YES** | **YES** | **YES** | **YES** |
| Khan et al^95^ | **YES** | **YES** | **YES** | **YES** | **NO** | **NO** | **YES** |
| Khanna et al^60^ | **YES** | **YES** | **NO** | **YES** | **YES** | **YES** | **YES** |
| Kibirige et al^117^ | **YES** | **YES** | **NO** | **YES** | **YES** | **YES** | **YES** |
| Paul et al^138^ | **YES** | **YES** | **YES** | **YES** | **YES** | **YES** | **YES** |
| Ko Picon et al^27^ | **YES** | **YES** | **YES** | **YES** | **YES** | **YES** | **YES** |
| Kumar et al^61^ | **YES** | **YES** | **YES** | **YES** | **YES** | **YES** | **YES** |
| KV et al^47^ | **YES** | **YES** | **NO** | **YES** | **YES** | **YES** | **YES** |
| Leal et al^6^ | **YES** | **YES** | **YES** | **YES** | **YES** | **YES** | **YES** |
| Lee et al^77^ | **YES** | **YES** | **YES** | **YES** | **NO** | **NO** | **YES** |
| Lee et al^78^ | **YES** | **YES** | **YES** | **YES** | **YES** | **YES** | **YES** |
| Leung et al^24^ | **YES** | **YES** | **YES** | **YES** | **YES** | **YES** | **YES** |
| Leung et al^30^ | **YES** | **YES** | **YES** | **YES** | **YES** | **YES** | **YES** |
| Loh et al^109^ | **NO** | **YES** | **NO** | **YES** | **NO** | **NO** | **YES** |
| Ma et al^15^ | **NO** | **YES** | **YES** | **YES** | **YES** | **YES** | **YES** |
| Magee et al^105^ | **YES** | **YES** | **YES** | **YES** | **YES** | **YES** | **YES** |
| Magee et al^121^ | **YES** | **YES** | **YES** | **YES** | **YES** | **YES** | **YES** |
| Magee et al^125^ | **YES** | **YES** | **YES** | **YES** | **YES** | **YES** | **YES** |
| Magee et al^126^ | **YES** | **YES** | **YES** | **YES** | **YES** | **YES** | **YES** |
| Mahishale et al^58^ | **NO** | **YES** | **YES** | **YES** | **NO** | **YES** | **YES** |
| Majumdar et al^143^ | **YES** | **YES** | **YES** | **YES** | **YES** | **YES** | **YES** |
| Manjareeka et al^51^ | **YES** | **YES** | **NO** | **YES** | **YES** | **NO** | **YES** |
| Mave et al^52^ | **YES** | **YES** | **YES** | **YES** | **YES** | **YES** | **YES** |
| Mburu et al^75^ | **YES** | **YES** | **YES** | **YES** | **YES** | **YES** | **YES** |
| Mehta et al^50^ | **YES** | **YES** | **NO** | **YES** | **YES** | **YES** | **YES** |
| Mi et al^12^ | **YES** | **YES** | **YES** | **YES** | **YES** | **YES** | **YES** |
| Mi Picon et al^23^ | **YES** | **YES** | **YES** | **YES** | **YES** | **YES** | **YES** |
| Gadallah et al^130^ | **YES** | **YES** | **YES** | **YES** | **YES** | **YES** | **YES** |
| Sane Schepisi et al^134^ | **YES** | **YES** | **YES** | **YES** | **YES** | **YES** | **YES** |
| Moreira et al^4^ | **YES** | **YES** | **NO** | **YES** | **YES** | **YES** | **YES** |
| Moreno-Martinez et al^110^ | **YES** | **YES** | **YES** | **YES** | **YES** | **YES** | **YES** |
| Mukhtar et al^101^ | **YES** | **YES** | **YES** | **YES** | **YES** | **YES** | **YES** |
| Munoz-Torrico et al^90^ | **YES** | **YES** | **NO** | **YES** | **YES** | **NO** | **YES** |
| Naik et al^57^ | **YES** | **YES** | **NO** | **YES** | **YES** | **YES** | **YES** |
| Nair et al^48^ | **YES** | **YES** | **YES** | **YES** | **YES** | **YES** | **YES** |
| Nakamura et al^71^ | **YES** | **YES** | **NO** | **YES** | **YES** | **NO** | **YES** |
| Nasa et al^84^ | **YES** | **YES** | **NO** | **YES** | **YES** | **YES** | **YES** |
| Omar et al^139^ | **YES** | **YES** | **YES** | **YES** | **YES** | **YES** | **YES** |
| Ogbera et al^97^ | **YES** | **YES** | **YES** | **YES** | **YES** | **YES** | **YES** |
| Ogbera et al^99^ | **YES** | **YES** | **YES** | **YES** | **YES** | **YES** | **YES** |
| Owiti et al^74^ | **NO** | **YES** | **YES** | **YES** | **YES** | **YES** | **YES** |
| Pande et al^42^ | **YES** | **YES** | **YES** | **YES** | **YES** | **YES** | **YES** |
| Park et al^80^ | **YES** | **YES** | **YES** | **YES** | **YES** | **YES** | **YES** |
| Pereira et al^8^ | **YES** | **YES** | **YES** | **YES** | **NO** | **YES** | **YES** |
| Perez-Navarro et al^86^ | **YES** | **YES** | **YES** | **YES** | **YES** | **YES** | **YES** |
| Picon et al^2^ | **NO** | **YES** | **YES** | **YES** | **NO** | **NO** | **YES** |
| Pizzol et al^94^ | **YES** | **YES** | **YES** | **YES** | **YES** | **YES** | **YES** |
| Ponce-De-Leon et al^92^ | **YES** | **YES** | **YES** | **YES** | **YES** | **YES** | **YES** |
| Prakash et al^49^ | **YES** | **YES** | **NO** | **YES** | **YES** | **YES** | **YES** |
| Prasad et al^34^ | **YES** | **YES** | **YES** | **YES** | **YES** | **YES** | **YES** |
| Qian et al^122^ | **YES** | **YES** | **YES** | **YES** | **NO** | **NO** | **YES** |
| Rajapakshe et al^111^ | **YES** | **YES** | **NO** | **YES** | **YES** | **YES** | **YES** |
| Reis-Santos et al^7^ | **YES** | **YES** | **YES** | **YES** | **YES** | **YES** | **YES** |
| Restrepo et al^119^ | **YES** | **YES** | **NO** | **YES** | **YES** | **YES** | **YES** |
| Saktiawati et al^62^ | **YES** | **YES** | **YES** | **YES** | **YES** | **YES** | **YES** |
| Salindri Gomes et al^38^ | **YES** | **YES** | **NO** | **YES** | **NO** | **YES** | **YES** |
| Sarker Gomes et al^32^ | **YES** | **YES** | **YES** | **YES** | **YES** | **YES** | **YES** |
| Sembiah et al^146^ | **YES** | **YES** | **YES** | **YES** | **YES** | **YES** | **YES** |
| Sahakyan et al^133^ | **YES** | **YES** | **YES** | **YES** | **YES** | **YES** | **YES** |
| Siddiqui et al^43^ | **YES** | **YES** | **NO** | **YES** | **YES** | **YES** | **YES** |
| Hameed et al^134^ | **YES** | **YES** | **NO** | **YES** | **YES** | **YES** | **YES** |
| Sreeramareddy et al^96^ | **YES** | **YES** | **NO** | **YES** | **NO** | **YES** | **YES** |
| Stalenhoef et al^64^ | **YES** | **YES** | **NO** | **YES** | **YES** | **NO** | **YES** |
| Suwanpimolkul et al^124^ | **YES** | **YES** | **YES** | **YES** | **YES** | **YES** | **YES** |
| Sulaiman et al^83^ | **YES** | **YES** | **YES** | **YES** | **YES** | **YES** | **YES** |
| Tchankam et al^9^ | **YES** | **YES** | **YES** | **YES** | **YES** | **YES** | **YES** |
| Tsao Picon et al^28^ | **YES** | **YES** | **YES** | **YES** | **NO** | **YES** | **YES** |
| Uchimura et al^72^ | **NO** | **YES** | **YES** | **YES** | **YES** | **YES** | **YES** |
| Veesa et al^53^ | **YES** | **YES** | **YES** | **YES** | **YES** | **NO** | **YES** |
| Venkatarathnamma et al ^54^ | **YES** | **YES** | **YES** | **YES** | **YES** | **YES** | **YES** |
| Viswanathan et al^45^ | **YES** | **YES** | **YES** | **YES** | **YES** | **YES** | **YES** |
| Viswanathan et al^56^ | **YES** | **YES** | **YES** | **YES** | **YES** | **YES** | **YES** |
| Nagar et al^145^ | **YES** | **YES** | **YES** | **YES** | **YES** | **YES** | **YES** |
| Walker et al^118^ | **YES** | **YES** | **YES** | **YES** | **NO** | **NO** | **YES** |
| Wang et al^13^ | **YES** | **YES** | **YES** | **YES** | **YES** | **YES** | **YES** |
| Wang et al^17^ | **YES** | **YES** | **YES** | **YES** | **NO** | **YES** | **YES** |
| Wang et al^18^ | **NO** | **YES** | **YES** | **YES** | **NO** | **NO** | **YES** |
| Wang et al^19^ | **YES** | **YES** | **YES** | **YES** | **YES** | **NO** | **YES** |
| Wang et al^20^ | **YES** | **YES** | **NO** | **YES** | **NO** | **NO** | **YES** |
| Wang et al^26^ | **YES** | **YES** | **NO** | **YES** | **NO** | **YES** | **YES** |
| Workneh et al^33^ | **YES** | **YES** | **YES** | **YES** | **YES** | **YES** | **YES** |
| Wu et al^14^ | **YES** | **YES** | **NO** | **YES** | **YES** | **YES** | **YES** |
| Yorke et al^39^ | **NO** | **YES** | **NO** | **YES** | **YES** | **YES** | **YES** |
| Yoon et al^79^ | **YES** | **YES** | **YES** | **YES** | **YES** | **YES** | **YES** |
| Pang et al^137^ | **YES** | **YES** | **YES** | **NO** | **YES** | **YES** | **YES** |
| Lee et al^148^ | **YES** | **YES** | **YES** | **YES** | **YES** | **YES** | **YES** |
| Hasan et al^131^ | **YES** | **YES** | **NO** | **YES** | **YES** | **YES** | **YES** |
| Araia et al^128^ | **YES** | **YES** | **YES** | **YES** | **YES** | **YES** | **YES** |
| Zhao et al^11^ | **YES** | **YES** | **NO** | **YES** | **YES** | **YES** | **YES** |
| Zhao et al^21^ | **YES** | **YES** | **YES** | **YES** | **YES** | **YES** | **YES** |
| Zhang et al^22^ | **YES** | **YES** | **YES** | **NO** | **NO** | **NO** | **YES** |

**Table S4**. The estimated prevalence of diabetes mellitus in patients with pulmonary tuberculosis, by country and WHO region

| Country | Prevalence (%) | 95% Confidence Interval | |
| --- | --- | --- | --- |
|  |  | Lower | Upper |
| **African Region (46)** | | | |
| Algeria | 11.72 | 2.50 | 40.77 |
| Angola | 6.61 | 0.36 | 58.07 |
| Benin | 6.42 | 0.28 | 57.26 |
| Botswana | 7.67 | 0.51 | 57.27 |
| Burkina Faso | 4.98 | 0.06 | 81.07 |
| Burundi | 7.24 | 0.23 | 72.7 |
| Cabo Verde | 7.13 | 0.77 | 43.10 |
| Cameroon* | 9.26 | 1.34 | 43.45 |
| Central African republic | 4.53 | 0.10 | 70.93 |
| Chad | 6.12 | 0.08 | 84.45 |
| Comoros | 18.35 | 0.49 | 91.10 |
| Congo Dem Rep | 6.31 | 0.41 | 52.42 |
| Congo Rep | 7.02 | 0.34 | 62.53 |
| Cote d’Ivoire | 5.78 | 0.47 | 44.41 |
| Equatorial Guinea | 9.57 | 1.27 | 46.52 |
| Eritrea | 7.41 | 0.23 | 74.07 |
| Eswatini | 6.34 | 0.48 | 48.94 |
| Ethiopia* | 9.06 | 0.62 | 61.41 |
| Gabon | 7.28 | 0.14 | 81.58 |
| Gambia | 4.75 | 0.18 | 58.84 |
| Ghana* | 8.39 | 1.44 | 36.45 |
| Guinea* | 5.78 | 0.30 | 55.28 |
| Guinea-Bissau | 4.94 | 0.20 | 57.85 |
| Kenya* | 37.21 | - | - |
| Lesotho | 3.90 | 0.04 | 82.06 |
| Liberia | 4.36 | 0.19 | 52.57 |
| Madagascar | 7.07 | 0.36 | 61.20 |
| Malawi | 7.01 | 0.36 | 61.20 |
| Mali | 4.86 | 0.05 | 50.91 |
| Mauritania | 6.76 | 0.51 | 50.91 |
| Mauritius | 41.59 | 4.02 | 92.38 |
| Mozambique* | 4.01 | 0.07 | 72.62 |
| Namibia | 4.87 | 0.06 | 80.19 |
| Niger | 7.01 | 0.38 | 59.94 |
| Nigeria* | 9.53 | - | - |
| Rwanda | 8.46 | 0.51 | 62.94 |
| Sao Tome and Principe | 6.41 | 0.69 | 40.33 |
| Senegal | 5.41 | 0.25 | 56.70 |
| Seychelles | 21.93 | 1.15 | 87.14 |
| Sierra Leone | 4.43 | 0.21 | 50.39 |
| South Africa* | 6.44 | 0.07 | 88.28 |
| Tanzania* | 16.38 | - | - |
| Togo | 9.29 | 0.51 | 67.03 |
| Uganda* | 5.58 | 0.53 | 40.11 |
| Zambia* | 5.70 | 0.40 | 47.84 |
| Zimbabwe | 4.83 | 0.18 | 58.34 |
| **Eastern Mediterranean Region (20)** | | | |
| Afghanistan | 10.09 | 0.73 | 63.31 |
| Bahrain | 32.81 | 5.85 | 79.32 |
| Djibouti | 6.61 | 0.26 | 65.75 |
| Egypt* | 27.80 | - | - |
| Iran* | 23.16 | - | - |
| Iraq | 12.92 | 1.57 | 58.08 |
| Jordan | 20.14 | 3.67 | 62.54 |
| Kuwait* | 27.27 | 4.58 | 74.54 |
| Lebanon | 22.74 | 3.37 | 71.32 |
| Libya | 18.87 | 1.61 | 76.81 |
| Morocco | 13.30 | 0.99 | 70.21 |
| Oman | 25.39 | 2.37 | 82.64 |
| Pakistan* | 5.02 | - | - |
| Qatar* | 29.83 | 5.91 | 74.19 |
| Saudi Arabia* | 8.24 | - | - |
| Sudan | 22.56 | 0.57 | 93.66 |
| Syrian Arab Republic | 10.48 | 0.33 | 80.38 |
| Tunisia | 15.72 | 2.88 | 53.99 |
| United Arab Emirates | 36.42 | 5.07 | 85.99 |
| Yemen | 7.93 | 0.31 | 70.18 |
| **European Region (50)** | | | |
| Albania | 16.81 | 2.75 | 59.12 |
| Andorra | 17.40 | 3.38 | 55.89 |
| Armenia | 15.73 | 1.06 | 76.41 |
| Austria | 16.23 | 2.13 | 63.30 |
| Azerbaijan | 14.08 | 2.28 | 53.47 |
| Belarus | 13.33 | 1.58 | 59.61 |
| Belgium | 13.91 | 1.26 | 67.09 |
| Bosnia and Herzegovina | 17.23 | 4.66 | 47.02 |
| Bulgaria | 13.89 | 2.02 | 55.72 |
| Croatia | 14.65 | 1.49 | 66.09 |
| Cyprus | 19.26 | 4.39 | 55.32 |
| Czech Republic | 15.38 | 3.06 | 51.18 |
| Denmark | 16.40 | 2.18 | 63.29 |
| Estonia | 11.60 | 1.75 | 49.15 |
| Finland | 14.98 | 2.20 | 58.03 |
| France* | 13.17 | 2.17 | 50.87 |
| Georgia* | 12.46 | 1.82 | 52.27 |
| Germany | 19.53 | 2.65 | 68.37 |
| Greece | 12.92 | 2.17 | 49.86 |
| Hungary | 14.61 | 2.28 | 55.62 |
| Iceland | 13.43 | 1.27 | 65.07 |
| Ireland | 12.00 | 1.23 | 59.95 |
| Israel | 15.89 | 2.86 | 54.81 |
| Italy* | 6.35 | 0.00 | 0.00 |
| Kazakhstan* | 13.05 | 2.52 | 46.59 |
| Kyrgyz Republic | 12.34 | 2.46 | 44.04 |
| Latvia | 12.39 | 2.47 | 44.09 |
| Lithuania | 11.64 | 1.62 | 51.29 |
| Luxembourg | 14.00 | 1.23 | 67.95 |
| Malta | 21.08 | 1.07 | 86.81 |
| Moldova | 12.44 | 1.41 | 58.59 |
| Montenegro | 20.16 | 3.59 | 63.13 |
| Netherlands | 16.16 | 0.80 | 82.19 |
| Norway | 15.62 | 1.43 | 70.31 |
| Poland | 15.06 | 1.96 | 61.14 |
| Portugal | 19.34 | 5.18 | 51.28 |
| Romania* | 17.02 | 5.03 | 44.25 |
| Russian Federation | 12.21 | 2.25 | 45.70 |
| Serbia | 19.70 | 3.51 | 62.33 |
| Slovak Republic | 17.88 | 1.07 | 81.49 |
| Slovenia | 17.91 | 1.93 | 70.76 |
| Spain* | 15.09 | 2.24 | 57.99 |
| Sweden | 14.95 | 1.20 | 71.83 |
| Switzerland | 16.57 | 1.04 | 78.99 |
| Tajikistan | 12.48 | 1.69 | 54.16 |
| Turkey* | 14.49 | 0.00 | 0.00 |
| Turkmenistan | 12.35 | 3.03 | 38.86 |
| Ukraine | 12.04 | 1.96 | 48.37 |
| United kingdom* | 11.11 | 0.00 | 0.00 |
| Uzbekistan | 13.74 | 2.66 | 48.15 |
| **Region of the Americas (35)** | | | |
| Antigua and Barbuda | 21.69 | 3.08 | 70.73 |
| Argentina* | 13.56 | 2.30 | 51.07 |
| Bahamas | 21.97 | 3.13 | 71.05 |
| Barbados | 26.92 | 3.24 | 80.23 |
| Belize | 27.78 | 3.34 | 81.06 |
| Bolivia | 11.20 | 2.79 | 35.64 |
| Brazil* | 6.55 | - | - |
| Canada | 16.28 | 2.64 | 58.19 |
| Chile | 16.71 | 4.53 | 45.88 |
| Colombia | 14.06 | 3.92 | 39.62 |
| Costa Rica | 17.53 | 3.68 | 54.17 |
| Cuba | 17.28 | 1.77 | 70.79 |
| Dominica | 21.24 | 2.53 | 73.68 |
| Dominican Republic | 15.86 | 2.27 | 60.45 |
| Ecuador | 12.74 | 1.42 | 59.60 |
| El Salvador | 13.28 | 2.46 | 48.19 |
| Grenada | 21.63 | 1.48 | 83.54 |
| Guatemala | 16.65 | 2.09 | 65.16 |
| Guyana* | 18.14 | 3.54 | 57.26 |
| Haiti | 7.40 | 0.30 | 68.21 |
| Honduras | 10.89 | 1.11 | 57.10 |
| Jamaica | 17.78 | 2.47 | 64.89 |
| Mexico* | 36.12 | - | - |
| Nicaragua | 14.49 | 0.14 | 95.29 |
| Panama | 16.14 | 4.50 | 44.05 |
| Paraguay | 14.31 | 3.80 | 41.34 |
| Peru | 3.11 | - | - |
| Saint Vincent and the Grenadines | 21.97 | 1.66 | 82.47 |
| St. Kitts and Nevis | 25.71 | 1.53 | 88.52 |
| St. Lucia | 19.16 | 3.55 | 60.43 |
| Suriname | 22.27 | 3.11 | 71.89 |
| Trinidad and Tobago | 19.60 | 5.51 | 50.46 |
| United states* | 15.48 | - | - |
| Uruguay | 14.78 | 3.04 | 48.96 |
| Venezuela | 12.31 | 2.53 | 43.17 |
| **South-East Asia Region (10)** | | | |
| Bangladesh* | 24.12 | 0.00 | 0.00 |
| Bhutan | 12.46 | 2.35 | 45.74 |
| India* | 16.06 | 0.00 | 0.00 |
| Indonesia* | 14.88 | 0.00 | 0.00 |
| Maldives | 15.80 | 4.23 | 44.39 |
| Myanmar* | 6.91 | 0.64 | 45.91 |
| Nepal* | 13.26 | 0.68 | 77.25 |
| Sri Lanka* | 19.99 | 2.59 | 70.15 |
| Thailand* | 12.57 | 3.17 | 38.71 |
| Timor-Leste | 7.94 | 0.22 | 77.43 |
| **Western pacific region (24)** | | | |
| Australia | 12.77 | 0.99 | 68.14 |
| Brunei Darussalam | 21.67 | 5.30 | 57.75 |
| Cambodia | 5.58 | 0.21 | 62.89 |
| China* | 12.00 | 0.00 | 0.00 |
| Fiji* | 14.20 | 0.00 | 0.00 |
| Japan* | 26.63 | 0.00 | 0.00 |
| Kiribati* | 28.97 | 3.73 | 81.12 |
| Korea, Rep* | 24.16 | 0.00 | 0.00 |
| Lao PDR | 8.30 | 0.80 | 50.31 |
| Malaysia* | 30.18 | 7.42 | 69.97 |
| Marshall Islands | 50.12 | 4.28 | 95.76 |
| Micronesia, Fed | 16.89 | 2.63 | 60.49 |
| Mongolia | 8.10 | 0.21 | 78.41 |
| Nauru | 40.89 | 1.80 | 96.31 |
| New Zealand | 12.31 | 1.23 | 61.38 |
| Palau | 29.03 | 7.15 | 68.48 |
| Papua New Guinea | 17.54 | 1.63 | 73.23 |
| Philippines | 7.47 | 0.13 | 83.25 |
| Samoa | 17.30 | 1.53 | 73.75 |
| Singapore* | 22.73 | 4.27 | 65.98 |
| Solomon Island | 31.11 | 0.61 | 97.06 |
| Tonga | 27.57 | 3.04 | 82.22 |
| Vanuatu | 19.22 | 0.94 | 85.70 |
| Vietnam |  |  |  |

*Estimation was based on the meta-analysis of the current literatures.

Note. Prediction of the prevalence of DM in patients with PTB for Democratic People’s Republic of Korea, Marshall Islands, Monaco, Nauru, San Marino, Somalia, South Sudan, Tuvalu was not available because of the missing data for the covariates.

**Table S5.** Results of the test of heterogeneity and publication bias for the meta-analysis of the prevalence of diabetes mellitus in patients with pulmonary tuberculosis, by country and WHO region

| **Country** | **Number of estimates** | **I^2^** | ***P*-value（regression test）** |
| --- | --- | --- | --- |
| **AFRICAN REGION** | | | |
| Kenya | 2 | - | 0.99 |
| Nigeria | 3 | - | 0.94 |
| Tanzania | 2 | - | 0.96 |
| **EASTERN MEDITERNEAN REGION** | | | |
| Egypt | 2 |  | 0.92 |
| Iran | 3 | - | 0.86 |
| Pakistan | 6 | - | 0.95 |
| Saudi Arabia | 2 | - | 0.89 |
| **EUROPEAN REGION** | | | |
| Italy | 2 |  | 0.98 |
| Turkey | 2 | - | 0.88 |
| **REGION OF THE AMERICAS** | | | |
| Mexico | 10 | - | 1.00 |
| Brazil | 7 | 93.2% | <0.001 |
| Peru | 3 | - | 0.82 |
| Unite States | 10 | - | 0.99 |
| **SOUTH-EAST ASIA REGION** | | | |
| Bangladesh | 2 |  | 0.88 |
| Indonesia | 5 | - | 0.99 |
| India | 28 | - | 1.00 |
| **WESTERN PACIFIC REGION** | | | |
| China | 24 | 21.7% | 0.17 |
| Fiji | 3 | - | 1.00 |
| Japan | 2 | - | 0.96 |
| Korea | 6 | - | 1.00 |

* The unequal numbers between estimates and studies were caused by some studies that reported more than one country’s prevalence of DM in patients with PTB.

**References**

1. Fernández R, Díaz A, D'Attilio L, Bongiovanni B, Santucci N, Bertola D*, et al.* An adverse immune-endocrine profile in patients with tuberculosis and type 2 diabetes. *Tuberculosis* 2016,**101**:95-101.

2. Picon PD, Bassanesi SL, Caramori ML, Ferreira RL, Jarczewski CA, Vieira PR. Risk factors for recurrence of tuberculosis. *J Bras Pneumol* 2007,**33**:572-578.

3. Gomes T, Reis-Santos B, Bertolde A, Johnson JL, Riley LW, Maciel EL. Epidemiology of extrapulmonary tuberculosis in Brazil: a hierarchical model. *BMC Infect Dis* 2014,**14**:9.

4. Moreira J, Castro R, Lamas C, Ribeiro S, Grinsztejn B, Veloso VG. Hyperglycemia during tuberculosis treatment increases morbidity and mortality in a contemporary cohort of HIV-infected patients in Rio de Janeiro, Brazil. *Int J Infect Dis* 2018,**69**:11-19.

5. Augusto CJ, Carvalho Wda S, Goncalves AD, Ceccato M, Miranda SS. Characteristics of tuberculosis in the state of Minas Gerais, Brazil: 2002-2009. *J Bras Pneumol* 2013,**39**:357-364.

6. Leal SB, Araujo GS, Nery JS, Santos C, Oliveira MG, Barreto ML*, et al.* Clinical and epidemiological aspects of cases of tuberculosis associated with diabetes in Salvador, Bahia, Brazil. *Rev Soc Bras Med Trop* 2017,**50**:408-412.

7. Reis-Santos B, Locatelli R, Horta BL, Faerstein E, Sanchez MN, Riley LW*, et al.* Socio-demographic and clinical differences in subjects with tuberculosis with and without diabetes mellitus in Brazil--a multivariate analysis. *PLoS One* 2013,**8**:e62604.

8. Pereira SM, Araujo GS, Santos CA, Oliveira MG, Barreto ML. Association between diabetes and tuberculosis: case-control study. *Rev Saude Publica* 2016,**50**:82.

9. Tchankam C, Pefura Yone EW, Nouedoui C, Kuaban C. Radio-clinical, biological and evolutional characteristics of pulmonary tuberculosis in patients with diabetes mellitus. *Diabetes Research and Clinical Practice* 2014,**103**:S18.

10. Chang LY, Lee CH, Chang CH, Lee MC, Lee MR, Wang JY*, et al.* Acute biliary events during anti-tuberculosis treatment: hospital case series and a nationwide cohort study. *BMC Infect Dis* 2018,**18**:64.

11. Zhao X, Yuan Y, Lin Y, Zhang T, Ma J, Kang W*, et al.* Vitamin D status in tuberculosis patients with diabetes, prediabetes and normal blood glucose in China: a cross-sectional study. *BMJ Open* 2017,**7**:e017557.

12. Mi F, Tan S, Liang L, Harries AD, Hinderaker SG, Lin Y*, et al.* Diabetes mellitus and tuberculosis: pattern of tuberculosis, two-month smear conversion and treatment outcomes in Guangzhou, China. *Trop Med Int Health* 2013,**18**:1379-1385.

13. Wang Q, Ma A, Han X, Zhao S, Cai J, Ma Y*, et al.* Prevalence of type 2 diabetes among newly detected pulmonary tuberculosis patients in China: a community based cohort study. *PLoS One* 2013,**8**:e82660.

14. Wu Z, Guo J, Huang Y, Cai E, Zhang X, Pan Q*, et al.* Diabetes mellitus in patients with pulmonary tuberculosis in an aging population in Shanghai, China: Prevalence, clinical characteristics and outcomes. *J Diabetes Complications* 2016,**30**:237-241.

15. Ma Y, Huang ML, Li T, Du J, Shu W, Xie SH, et al. Role of Diabetes Mellitus on Treatment Effects in Drug-susceptible Initial Pulmonary Tuberculosis Patients in China. Biomed Environ Sci 2017,30:671-675.

16. Huang L, Abe EM, Li XX, Bergquist R, Xu L, Xue JB*, et al.* Space-time clustering and associated risk factors of pulmonary tuberculosis in southwest China. *Infectious Diseases of Poverty* 2018,**7**.

17. Wang Q, Ma A, Han X, Zhao S, Cai J, Kok FJ*, et al.* Hyperglycemia is associated with increased risk of patient delay in pulmonary tuberculosis in rural areas. *J Diabetes* 2017,**9**:648-655.

18. Wang JY, Lee LN, Yu CJ, Chien YJ, Yang PC. Factors influencing time to smear conversion in patients with smear-positive pulmonary tuberculosis. *Respirology* 2009,**14**:1012-1019.

19. Wang JY, Lee LN, Hsueh PR. Factors changing the manifestation of pulmonary tuberculosis. *Int J Tuberc Lung Dis* 2005,**9**:777-783.

20. Wang CS, Chen HC, Yang CJ, Wang WY, Chong IW, Hwang JJ*, et al.* The impact of age on the demographic, clinical, radiographic characteristics and treatment outcomes of pulmonary tuberculosis patients in Taiwan. *Infection* 2008,**36**:335-340.

21. Zhao Q, Xiao X, Lu W, Qiu LX, Zhou CM, Jiang WL*, et al.* Screening diabetes in tuberculosis patients in eastern rural China: a community-based cross-sectional study. *Int J Tuberc Lung Dis* 2016,**20**:1370-1376.

22. Zhang Q, Xiao H, Sugawara I. Tuberculosis complicated by diabetes mellitus at shanghai pulmonary hospital, china. *Jpn J Infect Dis* 2009,**62**:390-391.

23. Mi F, Jiang G, Du J, Li L, Yue W, Harries AD*, et al.* Is resistance to anti-tuberculosis drugs associated with type 2 diabetes mellitus? A register review in Beijing, China. *Glob Health Action* 2014,**7**:24022.

24. Leung CC, Lam TH, Chan WM, Yew WW, Ho KS, Leung GM*, et al.* Diabetic control and risk of tuberculosis: a cohort study. *Am J Epidemiol* 2008,**167**:1486-1494.

25. Cai J, Ma A, Wang Q, Han X, Zhao S, Wang Y*, et al.* Association between body mass index and diabetes mellitus in tuberculosis patients in China: a community based cross-sectional study. *BMC Public Health* 2017,**17**:228.

26. Wang CS, Chen HC, Yang CJ, Tsai JR, Chong IW, Hwang JJ*, et al.* Clinical characteristics of pulmonary tuberculosis patients from a southern Taiwan hospital-based survey. *Kaohsiung J Med Sci* 2008,**24**:17-24.

27. Ko, Po-Yen, Hsu, Shang-Ren, Hsieh, Ming-Chia*, et al.* High diabetes mellitus prevalence with increasing trend among newly-diagnosed tuberculosis patients in an Asian population: A nationwide population-based study. *Primary care diabetes* 2016,**10**:148-155.

28. Tsao TC, Chiou W, Lin H, Wu T, Lin M, Yang P*, et al.* Change in demographic picture and increase of drug resistance in pulmonarytuberculosis in a 10-year interval in Taiwan. *Infection* 2002,**30**:75-80.

29. Chang JT, Dou HY, Yen CL, Wu YH, Huang RM, Lin HJ*, et al.* Effect of type 2 diabetes mellitus on the clinical severity and treatment outcome in patients with pulmonary tuberculosis: a potential role in the emergence of multidrug-resistance. *J Formos Med Assoc* 2011,**110**:372-381.

30. Leung CC, Yew WW, Mok TYW, Lau KS, Wong CF, Chau CH*, et al.* Effects of diabetes mellitus on the clinical presentation and treatment response in tuberculosis. *Respirology* 2017,**22**:1225-1232.

31. Hasanain AFA, Zayed AAAH, Mahdy RE, Nafee AMA, Attia RAMH, Mohamed AO. Hookworm infection among patients with pulmonary tuberculosis: Impact of co-infection on the therapeutic failure of pulmonary tuberculosis. *International Journal of Mycobacteriology* 2015,**4**:318-322.

32. Sarker M, Barua M, Guerra F, Saha A, Aftab A, Mahbub Latif AHM*, et al.* Double trouble: Prevalence and factors associated with tuberculosis and diabetes comorbidity in Bangladesh. *PLoS ONE* 2016,**11**

33. Workneh MH, Bjune GA, Yimer SA. Diabetes mellitus is associated with increased mortality during tuberculosis treatment: a prospective cohort study among tuberculosis patients in South-Eastern Amahra Region, Ethiopia. *Infect Dis Poverty* 2016,**5**:22.

34. Prasad P, Gounder S, Varman S, Viney K. Sputum smear conversion and treatment outcomes for tuberculosis patients with and without diabetes in Fiji. *Public Health Action* 2014,**4**:159-163.

35. Gounder S, Harries AD. Screening tuberculosis patients for diabetes mellitus in Fiji: notes from the field. *Public Health Action* 2012,**2**:145-147.

36. Alo A, Gounder S, Graham SM, Graham SM. Clinical characteristics and treatment outcomes of tuberculosis cases hospitalised in the intensive phase in Fiji. *Public Health Action* 2014,**4**:164-168.

37. Delory T, Ferrand H, Grall N, Casalino E, Lafarge M, Melot B*, et al.* Score for pulmonary tuberculosis in patients with clinical presumption of tuberculosis in a low-prevalence area. *International Journal of Tuberculosis and Lung Disease* 2017,**21**:1272-1279.

38. Salindri AD, Kipiani M, Kempker RR, Gandhi NR, Darchia L, Tukvadze N*, et al.* Diabetes reduces the rate of sputum culture conversion in patients with newly diagnosed multidrug-resistant tuberculosis. *Open Forum Infectious Diseases* 2016,**3**.

39. Yorke E, Boima V, Dey ID, Atiase Y. Examination of Dysglycaemia among Newly Diagnosed Tuberculosis Patients in Ghana: A Cross-Sectional Study. 2018,**2018**:1830372.

40. Haraldsdottir TL, Rudolf F, Bjerregaard-Andersen M, Joaquim LC, Stochholm K, Gomes VF*, et al.* Diabetes mellitus prevalence in tuberculosis patients and the background population in Guinea-Bissau: a disease burden study from the capital Bissau. *Trans R Soc Trop Med Hyg* 2015,**109**:400-407.

41. Alladin B, Mack S, Singh A, Singh C, Smith B, Cummings E*, et al.* Tuberculosis and diabetes in Guyana. *Int J Infect Dis* 2011,**15**:e818-821.

42. Pande T, Huddart S, Xavier W, Kulavalli S, Chen T, Pai M*, et al.* Prevalence of diabetes mellitus amongst hospitalized tuberculosis patients at an Indian tertiary care center: A descriptive analysis. *PLoS One* 2018,**13**:e0200838.

43. Siddiqui AN, Khayyam KU, Sharma M. Effect of Diabetes Mellitus on Tuberculosis Treatment Outcome and Adverse Reactions in Patients Receiving Directly Observed Treatment Strategy in India: A Prospective Study. *Biomed Res Int* 2016,**2016**:7273935.

44. Balakrishnan S, Vijayan S, Nair S, Subramoniapillai J, Mrithyunjayan S, Wilson N*, et al.* High diabetes prevalence among tuberculosis cases in Kerala, India. *PLoS One* 2012,**7**:e46502.

45. Viswanathan V, Kumpatla S, Aravindalochanan V, Rajan R, Chinnasamy C, Srinivasan R*, et al.* Prevalence of diabetes and pre-diabetes and associated risk factors among tuberculosis patients in India. *PLoS One* 2012,**7**:e41367.

46. Dave P, Shah A, Chauhan M, Kumar AM, Harries AD, Malhotra S*, et al.* Screening patients with tuberculosis for diabetes mellitus in Gujarat, India. *Public Health Action* 2013,**3**:S29-33.

47. K VN, Duraisamy K, Balakrishnan S, M S, S JS, Sagili KD*, et al.* Outcome of tuberculosis treatment in patients with diabetes mellitus treated in the revised national tuberculosis control programme in Malappuram District, Kerala, India. *PLoS One* 2013,**8**:e76275.

48. Nair S, Kumari AK, Subramonianpillai J, Shabna DS, Kumar SM, Balakrishnan S*, et al.* High prevalence of undiagnosed diabetes among tuberculosis patients in peripheral health facilities in Kerala. *Public Health Action* 2013,**3**:S38-42.

49. Prakash BC, Ravish KS, Prabhakar B, Ranganath TS, Naik B, Satyanarayana S*, et al.* Tuberculosis-diabetes mellitus bidirectional screening at a tertiary care centre, south india. *Public Health Action* 2013,**3**:S18-S22.

50. Mehta S, Yu EA, Ahamed SF, Bonam W, Kenneth J. Rifampin resistance and diabetes mellitus in a cross-sectional study of adult patients in rural South India. *BMC Infect Dis* 2015,**15**:451.

51. Manjareeka M, Palo SK, Swain S, Pati S, Pati S. Diabetes Mellitus among Newly Diagnosed Tuberculosis Patients in Tribal Odisha: An Exploratory Study. *J Clin Diagn Res* 2016,**10**:Lc06-lc08.

52. Mave V, Meshram S, Lokhande R, Kadam D, Dharmshale S, Bharadwaj R*, et al.* Prevalence of dysglycemia and clinical presentation of pulmonary tuberculosis in Western India. *Int J Tuberc Lung Dis* 2017,**21**:1280-1287.

53. Veesa KS, John KR, Moonan PK, Kaliappan SP, Manjunath K, Sagili KD*, et al.* Diagnostic pathways and direct medical costs incurred by new adult pulmonary tuberculosis patients prior to anti-tuberculosis treatment - Tamil Nadu, India. *PLoS ONE* 2018,**13**.

54.

55. Gupte AN, Mave V, Meshram S, Lokhande R, Kadam D, Dharmshale S*, et al.* Trends in HbA1c levels and implications for diabetes screening in tuberculosis cases undergoing treatment in India. *Int J Tuberc Lung Dis* 2018(55）,**22**:800-806.

56. Viswanathan V, Vigneswari A, Selvan K, Satyavani K, Rajeswari R, Kapur A. Effect of diabetes on treatment outcome of smear-positive pulmonary tuberculosis--a report from South India. *J Diabetes Complications* 2014,**28**:162-165.

57. Naik B, Kumar AM, Satyanarayana S, Suryakant MD, Swamy NM, Nair S*, et al.* Is screening for diabetes among tuberculosis patients feasible at the field level? *Public Health Action* 2013,**3**:S34-37.

58. Mahishale V, Avuthu S, Patil B, Lolly M, Eti A, Khan S. Effect of Poor Glycemic Control in Newly Diagnosed Patients with Smear-Positive Pulmonary Tuberculosis and Type-2 Diabetes Mellitus. *Iran J Med Sci* 2017,**42**:144-151.

59. Gupta S, Shenoy VP, Bairy I, Srinivasa H, Mukhopadhyay C. Diabetes mellitus and HIV as co-morbidities in tuberculosis patients of rural south India. *J Infect Public Health* 2011,**4**:140-144.

60. Khanna A, Lohya S, Sharath BN, Harries AD. Characteristics and treatment response in patients with tuberculosis and diabetes mellitus in New Delhi, India. *Public Health Action* 2013,**3**:S48-50.

61. Kumar AKH, Chandrasekaran V, Kannan T, Murali AL, Lavanya J, Sudha V*, et al.* Anti-tuberculosis drug concentrations in tuberculosis patients with and without diabetes mellitus. *European Journal of Clinical Pharmacology* 2017,**73**:65-70.

62. Saktiawati AMI, Subronto YW. Influence of Diabetes Mellitus on the Development of Multi Drug Resistant-Tuberculosis in Yogyakarta. *Acta Med Indones* 2018,**50**:11-17.

63. Alisjahbana B, Sahiratmadja E, Nelwan EJ, Purwa AM, Ahmad Y, Ottenhoff TH*, et al.* The effect of type 2 diabetes mellitus on the presentation and treatment response of pulmonary tuberculosis. *Clin Infect Dis* 2007,**45**:428-435.

64. Stalenhoef JE, Alisjahbana B, Nelwan EJ, van der Ven-Jongekrijg J, Ottenhoff TH, van der Meer JW*, et al.* The role of interferon-gamma in the increased tuberculosis risk in type 2 diabetes mellitus. *Eur J Clin Microbiol Infect Dis* 2008,**27**:97-103.

65. Fachri M, Abadi S. AFB smear M.TB on adult pulmonary TB patients with type 2 DM and without: A cross sectional study in general hospital North Jakarta Indonesia. *Respirology* 2017(65）,**22**:33.

66. Grint D, Alisjhabana B, Ugarte-Gil C, Riza AL, Walzl G, Pearson F*, et al.* Accuracy of diabetes screening methods used for people with tuberculosis, Indonesia, Peru, Romania, South Africa. *Bull World Health Organ* 2018,**96**:738-749.

67. Alavi-Naini R, Moghtaderi A, Metanat M, Mohammadi M, Zabetian M. Factors associated with mortality in tuberculosis patients. *J Res Med Sci* 2013,**18**:52-55.

68. Golsha R, Rezaei SR, Shafiee A, Najafi L, Dashti M, Roshandel G. Pulmonary tuberculosis and some underlying conditions in Golestan Province of Iran, during 2001-2005. *Journal of Clinical and Diagnostic Research* 2009,**3**:1302-1306.

69. Alavi SM, Khoshkho MM, Salmanzadeh S, Eghtesad M. Comparison of epidemiological, clinical,laboratory and radiological features of hospitalized diabetic and non-diabetic patients with pulmonary tuberculosis at razi hospital in ahvaz. *Jundishapur J Microbiol* 2014,**7**:e12447.

70. Caraffa E, Sañe Schepisi M, Gualano G, Parracino MP, Rianda A, Corpolongo A*, et al.* The diabetes-tuberculosis co-epidemic: The role of international migration. *International Journal of Tuberculosis and Lung Disease* 2016,**20**:771-777.

71. Nakamura A, Hagiwara E, Hamai J, Taguri M, Terauchi Y. Impact of underlying diabetes and presence of lung cavities on treatment outcomes in patients with pulmonary tuberculosis. *Diabet Med* 2014,**31**:707-713.

72. Uchimura K, Ngamvithayapong-Yanai J, Kawatsu L, Ohkado A, Yoshiyama T, Shimouchi A*, et al.* Characteristics and treatment outcomes of tuberculosis cases by risk groups, Japan, 2007-2010. *Western Pac Surveill Response J* 2013,**4**:11-18.

73. Hermosilla S, You P, Aifah A, Abildayev T, Akilzhanova A, Kozhamkulov U*, et al.* Identifying risk factors associated with smear positivity of pulmonary tuberculosis in Kazakhstan. *PLoS One* 2017,**12**:e0172942.

74. Owiti P, Keter A, Harries AD, Pastakia S, Wambugu C, Kirui N*, et al.* Diabetes and pre-diabetes in tuberculosis patients in western Kenya using point-of-care glycated haemoglobin. *Public Health Action* 2017,**7**:147-154.

75. Mburu JW, Kingwara L, Ester M, Andrew N. Prognostic factors among TB and TB/DM comorbidity among patients on short course regimen within Nairobi and Kiambu counties in Kenya. *Journal of Clinical Tuberculosis and Other Mycobacterial Diseases* 2018,**12**:9-13.

76. Cavanaugh J, Viney K, Kienene T, Harley D, Kelly PM, Sleigh A*, et al.* Effect of diabetes on tuberculosis presentation and outcomes in Kiribati. *Trop Med Int Health* 2015,**20**:643-649.

77. Lee YJ, Han SK, Park JH, Lee JK, Kim DK, Chung HS*, et al.* The effect of metformin on culture conversion in tuberculosis patients with diabetes mellitus. *Korean J Intern Med* 2018,**33**:933-940.

78. Lee, Hye E, Lee, Mo J, Kang, Ae Y*, et al.* Prevalence and Impact of Diabetes Mellitus Among Patients with Active Pulmonary Tuberculosis in South Korea. *Lung* 2017,**195**:209-215.

79. Yoon YS, Jung JW, Jeon EJ, Seo H, Ryu YJ, Yim JJ*, et al.* The effect of diabetes control status on treatment response in pulmonary tuberculosis: a prospective study. *Thorax* 2017,**72**:263-270.

80. Park SW, Shin JW, Kim JY, Park IW, Choi BW, Choi JC*, et al.* The effect of diabetic control status on the clinical features of pulmonary tuberculosis. *Eur J Clin Microbiol Infect Dis* 2012,**31**:1305-1310.

81. Choi H, Lee M, Chen RY, Kim Y, Yoon S, Joh JS*, et al.* Predictors of pulmonary tuberculosis treatment outcomes in South Korea: a prospective cohort study, 2005-2012. *BMC Infect Dis* 2014,**14**:360.

82. Abal AT, Jayakrishnan B, Parwer S, El Shamy AS, Khadadah M, Ayed A*, et al.* Demographic pattern and clinical characteristics of patients with smear- positive pulmonary tuberculosis in kuwait. *Med Princ Pract* 2005,**14**:306-312.

83. Sulaiman SAS, Khan AH, Muttalif AR, Hassali MA, Ahmad N, Iqubal MS. Impact of diabetes mellitus on treatment outcomes of tuberculosis patients in tertiary care setup. *American Journal of the Medical Sciences* 2013,**345**:321-325.

84. Nasa JN, Brostrom R, Ram S, Kumar AMV, Seremai J, Hauma M*, et al.* Screening adult tuberculosis patients for diabetes mellitus in ebeye, republic of the Marshall Islands. *Public Health Action* 2014,**4**:S50-S52.

85. Blanco-Guillot F, Castaheda-Cediel ML, Cruz-Hervert P, Ferreyra-Reyes L, Delgado-Sanchez G, Ferreira-Guerrero E*, et al.* Genotyping and spatial analysis of pulmonary tuberculosis and diabetes cases in the state of Veracruz, Mexico. *PLoS ONE* 2018,**13**.

86. Perez-Navarro LM, Restrepo BI, Fuentes-Dominguez FJ, Duggirala R, Morales-Romero J, López-Alvarenga JC*, et al.* The effect size of type 2 diabetes mellitus on tuberculosis drug resistance and adverse treatment outcomes. *Tuberculosis* 2017,**103**:83-91.

87. Chittoor G, Arya R, Farook VS, David R, Puppala S, Resendez RG*, et al.* Epidemiologic investigation of tuberculosis in a Mexican population from Chihuahua State, Mexico: a pilot study. *Tuberculosis (Edinb)* 2013,**93 Suppl**:S71-77.

88. Jiménez-Corona ME, Cruz-Hervert LP, García-García L, Ferreyra-Reyes L, Delgado-Sánchez G, Bobadilla-Del-Valle M*, et al.* Association of diabetes and tuberculosis: Impact on treatment and post-treatment outcomes. *Thorax* 2013,**68**:214-220.

89. Castellanos-Joya M, Delgado-Sanchez G, Ferreyra-Reyes L, Cruz-Hervert P, Ferreira-Guerrero E,Ortiz-Solis G*, et al.* Results of the implementation of a pilot model for the bidirectional screening and joint management of patients with pulmonary tuberculosis and diabetes mellitus in Mexico. *PLoS One* 2014,**9**:e106961.

90. Munoz-Torrico M, Caminero-Luna J, Migliori GB, D'Ambrosio L, Carrillo-Alduenda JL, Villareal-Velarde H*, et al.* Diabetes is Associated with Severe Adverse Events in Multidrug-Resistant Tuberculosis. *Arch Bronconeumol* 2017,**53**:245-250.

91. Abdelbary BE, Garcia-Viveros M, Ramirez-Oropesa H, Rahbar MH, Restrepo BI. Tuberculosis-diabetes epidemiology in the border and non-border regions of Tamaulipas, Mexico. *Tuberculosis (Edinb)* 2016,**101s**:S124-s134.

92. Ponce-De-Leon A, Garcia-Garcia Md Mde L, Garcia-Sancho MC, Gomez-Perez FJ, Valdespino-Gomez JL, Olaiz-Fernandez G*, et al.* Tuberculosis and diabetes in southern Mexico. *Diabetes Care* 2004,**27**:1584-1590.

93. Delgado-Sánchez G, García-García L, Castellanos-Joya M, Cruz-Hervert P, Ferreyra-Reyes L, Ferreira-Guerrero E*, et al.* Association of pulmonary tuberculosis and diabetes in Mexico: Analysis of the National Tuberculosis Registry 2000-2012. *PLoS ONE* 2015,**10**.

94. Pizzol D, Di Gennaro F, Chhaganlal KD, Fabrizio C, Monno L, Putoto G*, et al.* Prevalence of diabetes mellitus in newly diagnosed pulmonary tuberculosis in Beira, Mozambique. *Afr Health Sci* 2017,**17**:773-779.

95. Khan MS, Hutchison C, Coker RJ. Risk factors that may be driving the emergence of drug resistance in tuberculosis patients treated in Yangon, Myanmar. *PLoS ONE* 2017,**12**.

96. Sreeramareddy CT, Panduru KV, Verma SC, Joshi HS, Bates MN. Comparison of pulmonary and extrapulmonary tuberculosis in Nepal - A hospital-based retrospective study. *BMC Infectious Diseases* 2008,**8**.

97. Ogbera AO, Kapur A, Odeyemi K, Longe-Peters K, Adeyeye OO, Odeniyi I*, et al.* Screening for diabetes mellitus and human immunodefiency virus infection in persons with tuberculosis. *J Prev Med Hyg* 2014,**55**:42-45.

98. Ekeke N, Ukwaja KN, Chukwu JN, Nwafor CC, Meka AO, Egbagbe EE*, et al.* Screening for diabetes mellitus among tuberculosis patients in Southern Nigeria: a multi-centre implementation study under programme settings. *Sci Rep* 2017,**7**:44205.

99. Ogbera AO, Kapur A, Abdur-Razzaq H, Harries AD, Ramaiya K, Adeleye O*, et al.* Clinical profile of diabetes mellitus in tuberculosis. *BMJ Open Diabetes Res Care* 2015,**3**:e000112.

100. Aftab H, Ambreen A, Jamil M, Garred P, Petersen JH, Nielsen SD*, et al.* High prevalence of diabetes and anthropometric heterogeneity among tuberculosis patients in Pakistan. *Trop Med Int Health* 2017,**22**:465-473.

101. Mukhtar F, Butt ZA. Cohort profile: the diabetes-tuberculosis treatment outcome (DITTO) study in Pakistan. *BMJ Open* 2016,**6**:e012970.

102. Jawad F, Shera AS, Memon R, Ansari G. Glucose intolerance in pulmonary tuberculosis. *J Pak Med Assoc* 1995,**45**:237-238.

103. Aftab H, Ambreen A, Jamil M, Garred P, Petersen JH, Nielsen SD*, et al.* Comparative study of HbA1c and fasting plasma glucose vs the oral glucose tolerance test for diagnosis of diabetes in people with tuberculosis. *Diabet Med* 2017,**34**:800-803.

104. Byrne AL, Marais BJ, Mitnick CD, Garden FL, Lecca L, Contreras C*, et al.* Feasibility and yield of screening for non-communicable diseases among treated tuberculosis patients in Peru. *Int J Tuberc Lung Dis* 2018,**22**:86-92.

105. Magee MJ, Bloss E, Shin SS, Contreras C, Huaman HA, Ticona JC*, et al.* Clinical characteristics, drug resistance, and treatment outcomes among tuberculosis patients with diabetes in Peru. *Int J Infect Dis* 2013,**17**:e404-412.

106. Al-Shaer MH, Mansour H, Elewa H, Salameh P, Iqbal F. Treatment outcomes of fixed-dose combination versus separate tablet regimens in pulmonary tuberculosis patients with or without diabetes in Qatar. *BMC Infect Dis* 2017,**17**:118.

107. Alkabab YM, Enani MA, Indarkiri NY, Heysell SK. Performance of computed tomography versus chest radiography in patients with pulmonary tuberculosis with and without diabetes at a tertiary hospital in Riyadh, Saudi Arabia. *Infect Drug Resist* 2018,**11**:37-43.

108. Chaudhry LA, Essa EB, Al-Solaiman S, Al-Sindi K. Prevalence of diabetes type-2 & pulmonary tuberculosis among Filipino and treatment outcomes: A surveillance study in the Eastern Saudi Arabia. *Int J Mycobacteriol* 2012,**1**:106-109.

109. Loh WJ, Yu Y, Loo CM, Low SY. Factors associated with mortality among patients with active pulmonary tuberculosis requiring intensive care. *Singapore Med J* 2017,**58**:656-659.

110. Moreno-Martinez A, Casals M, Orcau A, Gorrindo P, Masdeu E, Cayla JA. Factors associated with diabetes mellitus among adults with tuberculosis in a large European city, 2000-2013. *Int J Tuberc Lung Dis* 2015,**19**:1507-1512.

111. Rajapakshe W, Isaakidis P, Sagili KD, Kumar AMV, Samaraweera S, Pallewatta N*, et al.* Screening patients with tuberculosis for diabetes mellitus in Ampara, Sri Lanka. *Public Health Action* 2015,**5**:150-152.

112. Faurholt-Jepsen D, Range N, PrayGod G, Jeremiah K, Faurholt-Jepsen M, Aabye MG*, et al.* The role of diabetes on the clinical manifestations of pulmonary tuberculosis. *Trop Med Int Health* 2012,**17**:877-883.

113. Faurholt-Jepsen D, Range N, Praygod G, Jeremiah K, Faurholt-Jepsen M, Aabye MG*, et al.* Diabetes is a risk factor for pulmonary tuberculosis: a case-control study from Mwanza, Tanzania. *PLoS One* 2011,**6**:e24215.

114. Duangrithi D, Thanachartwet V, Desakorn V, Jitruckthai P, Phojanamongkolkij K, Rienthong S*, et al.* Impact of diabetes mellitus on clinical parameters and treatment outcomes of newly diagnosed pulmonary tuberculosis patients in Thailand. *Int J Clin Pract* 2013,**67**:1199-1209.

115. Bacakoglu F, Basoglu OK, Cok G, Sayiner A, Ates M. Pulmonary tuberculosis in patients with diabetes mellitus. *Respiration* 2001,**68**:595-600.

116. Guler M, Unsal E, Dursun B, Aydln O, Capan N. Factors influencing sputum smear and culture conversion time among patients with new case pulmonary tuberculosis. *Int J Clin Pract* 2007,**61**:231-235.

117. Kibirige D, Ssekitoleko R, Mutebi E, Worodria W. Overt diabetes mellitus among newly diagnosed Ugandan tuberculosis patients: a cross sectional study. *BMC Infect Dis* 2013,**13**:122.

118. Walker C, Unwin N. Estimates of the impact of diabetes on the incidence of pulmonary tuberculosis in different ethnic groups in England. *Thorax* 2010,**65**:578-581.

119. Restrepo BI, Camerlin AJ, Rahbar MH, Wang W, Restrepo MA, Zarate I*, et al.* Cross-sectional assessment reveals high diabetes prevalence among newly-diagnosed tuberculosis cases. *Bulletin of the World Health Organization* 2011,**89**:352-359.

120. Dooley KE, Tang T, Golub JE, Dorman SE, Cronin W. Impact of diabetes mellitus on treatment outcomes of patients with active tuberculosis. *Am J Trop Med Hyg* 2009,**80**:634-639.

121. Magee MJ, Kempker RR, Kipiani M, Gandhi NR, Darchia L, Tukvadze N*, et al.* Diabetes mellitus is associated with cavities, smear grade, and multidrug-resistant tuberculosis in Georgia. *Int J Tuberc Lung Dis* 2015,**19**:685-692.

122. Qian X, Nguyen DT, Lyu J, Albers AE, Bi X, Graviss EA. Risk factors for extrapulmonary dissemination of tuberculosis and associated mortality during treatment for extrapulmonary tuberculosis article. *Emerging Microbes and Infections* 2018,**7**.

123. Alkabab Y, Keller S, Dodge D, Houpt E, Staley D, Heysell S. Early interventions for diabetes related tuberculosis associate with hastened sputum microbiological clearance in Virginia, USA. *BMC Infect Dis* 2017,**17**:125.

124. Suwanpimolkul G, Grinsdale JA, Jarlsberg LG, Higashi J, Osmond DH, Hopewell PC*, et al.* Association between diabetes mellitus and tuberculosis in United States-born and foreign-born populations in San Francisco. *PLoS One* 2014,**9**:e114442.

125. Magee MJ, Foote M, Ray SM, Gandhi NR, Kempker RR. Diabetes mellitus and extrapulmonary tuberculosis: site distribution and risk of mortality. *Epidemiol Infect* 2016,**144**:2209-2216.

126. Magee MJ, Kempker RR, Kipiani M, Tukvadze N, Howards PP, Narayan KM*, et al.* Diabetes mellitus, smoking status, and rate of sputum culture conversion in patients with multidrug-resistant tuberculosis: a cohort study from the country of Georgia. *PLoS One* 2014,**9**:e94890.

127. Fwoloshi S, Hachaambwa LM, Chiyeñu KO, Chirwa L, Hoffman TW, Ngalamika O*, et al.* Screening for Diabetes Mellitus among Tuberculosis Patients: Findings from a Study at a Tertiary Hospital in Lusaka, Zambia. *Canadian Journal of Infectious Diseases and Medical Microbiology* 2018,**2018**.

128. Segafredo, G., et al., Integrating TB and non-communicable diseases services: Pilot experience of screening for diabetes and hypertension in patients with Tuberculosis in Luanda, Angola. PLoS One, 2019. 14(7).

129. Araia, Z.Z., et al., Diabetes Mellitus and Its Associated Factors in Tuberculosis Patients in Maekel Region, Eritrea: Analytical Cross-Sectional Study. Diabetes Metab Syndr Obes, 2021.

130. Gadallah, M., et al., Screening for diabetes among tuberculosis patients: a nationwide population-based study in Egypt. Afr Health Sci, 2018. 18(4).

131. Hasan, Z., et al., Raised levels of IFN-gamma and IL-13 are associated with pre-diabetes amongst newly diagosed patients with tuberculosis. Journal of the Pakistan Medical Association, April 2019. 69(4).

132. Hameed, S., et al., Risk factors for mortality among inpatients with smear positive pulmonary tuberculosis. Pak J Med Sci, 2019. 35(5).

133. Sahakyan, S., V. Petrosyan, and L. Abrahamyan, Diabetes mellitus and treatment outcomes of pulmonary tuberculosis: a cohort study. Int J Public Health, 2020. 65(1).

134. Sane Schepisi, M., et al., Burden and Characteristics of the Comorbidity Tuberculosis-Diabetes in Europe: TBnet Prevalence Survey and Case-Control Study. Open Forum Infect Dis, 2019.

135. Kreisel, C.F., M.R. Passannante, and A.A. Lardizabal, The Negative Clinical Impact of Diabetes on Tuberculosis: A Cross-Sectional Study in New Jersey. J Endocr Soc, 2019. 3(1): p. 62-68.

136. Nguyen, D.T. and E.A. Graviss, Diabetic trends and associated mortality in tuberculosis patients in Texas, a large population-based analysis. Tuberculosis (Edinb), 2019.

137. Pang, Y., et al., Epidemiology of Extrapulmonary Tuberculosis among Inpatients, China, 2008-2017. Emerg Infect Dis, 2019. 25(3).

138. Paul, K.K., et al., A public-private model to scale up diabetes mellitus screening among people accessing tuberculosis diagnostics in Dhaka, Bangladesh. Int J Infect Dis, 2020.

139. Omar, N., et al., Prevalence and associated factors of diabetes mellitus among tuberculosis patients in Brunei Darussalam: A 6-year retrospective cohort study. Int J Infect Dis, 2021.

140. Sharma, D., et al., Prevalence of Diabetes Mellitus and its Predictors among Tuberculosis Patients Currently on Treatment. Indian J Community Med, 2018. 43(4): p. 302-306.

141. Krishnappa, D., et al., Impact of tuberculosis on glycaemic status: A neglected association. Indian J Med Res, 2019. 149(3): p. 384-388.

142. Deshmukh, S., et al., Assessment of the Xpert assay among adult pulmonary tuberculosis suspects with and without diabetes mellitus. Int J Tuberc Lung Dis, 2020. 24(1): p. 113-117.

143. Majumdar, A., et al., Tuberculosis-diabetes screening: how well are we doing? A mixed-methods study from North India. Public Health Action, 2019. 9(1).

144. Christopher, D.J., et al., Burden of diabetes among patients with tuberculosis: 10-year experience from a tertiary care referral teaching hospital in South India. Lung India, 2020. 37(3).

145. Nagar, V., et al., Screening for diabetes among tuberculosis patients registered under revised national tuberculosis control program, Bhopal, India. J Family Med Prim Care, 2018. 7(6).

146. Sembiah, S., et al., Diabetes in tuberculosis patients: An emerging public health concern and the determinants and impact on treatment outcome. J Family Community Med, 2020. 27(2).

147. Kodiatte, A., M. John, and J.J. Jacob, Diabetes mellitus and prediabetes among patients with tuberculosis in a single north Indian tertiary care centre. J R Coll Physicians Edinb, 2020. 50(3).

148. Lee, Y.J., et al., The effect of metformin on culture conversion in tuberculosis patients with

diabetes mellitus. Korean J Intern Med, 2018. 33(5).

149. Hoa, N.B., et al., Prevalence and associated factors of diabetes mellitus among tuberculosis patients in Hanoi, Vietnam. BMC Infect Dis, 2018. 18(1).
